# Alcohol-Drinking Under Limited-Access Procedures During Mature Adulthood Accelerates the Onset of Cognitive Impairment in Mice

**DOI:** 10.3389/fnbeh.2022.732375

**Published:** 2022-05-24

**Authors:** C. Leonardo Jimenez Chavez, Eliyana Van Doren, Jacob Matalon, Nneoma Ogele, Aadithya Kharwa, Lauren Madory, Ida Kazerani, Jessica Herbert, Jose Torres-Gonzalez, Emely Rivera, Karen K. Szumlinski

**Affiliations:** ^1^Department of Psychological and Brain Sciences, University of California, Santa Barbara, Santa Barbara, CA, United States; ^2^Department of Molecular, Cellular and Developmental Biology and the Neuroscience Research Institute, University of California, Santa Barbara, Santa Barbara, CA, United States

**Keywords:** binge-drinking, working memory, spatial memory, aging, sex differences, dementia

## Abstract

A history of heavy drinking increases vulnerability to, and the severity of, Alzheimer’s disease (AD) and related dementias, with alcohol use disorder identified as the strongest modifiable risk factor for early-onset dementia. Heavy drinking has increased markedly in women over the past 10 years, particularly in mature adult women during the coronavirus (COVID-19) pandemic. This is concerning as women are more sensitive to many alcohol-related disease states, including AD and related dementias. Herein, we conducted two studies to determine if a 1-month period of binge drinking during mature adulthood (i.e., 5–9 months of age) impairs spatial and working memory to a greater extent in female vs. male C57BL/6J (B6J) mice. The anxiogenic and cognitive-impairing effects of binge drinking were also compared between mature adult and old B6J mice (18 months of age) in a third study. Throughout, females consumed more alcohol than males, indicating that a sex difference in binge drinking persists into old age. Despite the sex difference in intake, we detected no consistent sex difference in our measures of alcohol withdrawal-induced anxiety during a behavioral test battery. Although mature adult females exhibited more cognitive deficits than males, the precise outcome exhibiting a female-selective effect varied across studies. Old mice drank lower amounts of alcohol than mature adult mice, yet their blood ethanol concentrations (BECs) were within error of the 80 mg/dl criterion for binge drinking, indicative of an age-related slowing of alcohol metabolism. As expected, 18-month-old controls exhibited more signs of cognitive impairment than their 6-month-old counterparts, and binge drinking history impaired the Morris water maze performance of mice of both ages. In contrast, binge drinking history impaired the radial arm maze performance of 6-month-old mice only, and the extent of the impairment was comparable to the behavior exhibited by the older mice. We conclude from our studies that: (1) both biological sex and the age of drinking onset are subject factors that impact voluntary alcohol consumption by mice into old age; (2) binge drinking during later life elicits a negative affective state that is relatively sex-independent; (3) binge drinking during both mature adulthood and old age impairs spatial learning and memory; (4) binge drinking during mature adulthood accelerates deficits in working memory; and (5) mature adult females tend to exhibit more alcohol-induced cognitive impairments than males. If relevant to humans, these findings suggest that binge-like drinking by older adult men and women induces a negative affective state and cognitive decline, but that mature adult women, in particular, may be more sensitive to both the immediate and persistent cognitive-impairing effects of heavy drinking.

## Introduction

Alcohol use disorder (AUD) and dementia, including Alzheimer’s disease (AD) have a high rate of comorbidity (Thomas and Rockwood, 2001; McMurtray et al., 2006; Schwarzinger et al., 2018; Nunes et al., 2019) that is observed across nearly all sociodemographic groups (Grant et al., 2017). Even light-to-moderate alcohol consumption (<8 drinks/week) is associated with cognitive decline (e.g., Topiwala et al., 2017), while heavy alcohol drinking (<14 drinks/week) is reported to significantly increase the likelihood of developing dementia (Weyerer et al., 2011; Xu et al., 2017; Huang et al., 2018; Sabia et al., 2018). Excessive alcohol consumption is reported to reduce the age of dementia-onset in humans (Ledesma et al., 2020), as well as in laboratory rodents (e.g., Crews and Vetreno, 2014; Hoffman et al., 2019). Epidemiological data identify AUDs as the strongest modifiable risk factor for dementia onset, accounting for approximately 60% of early-onset dementia cases (Schwarzinger et al., 2018). Although AUD is more prevalent in men, the prevalence of AUD in women has increased by 84% over the past 10 years, compared to a 35% increase in men (Peltier et al., 2019). This trend is even more concerning considering that women are nearly twice as likely to develop AD and related dementias than men (Hebert et al., 2013; Ferretti et al., 2018).

Globally, binge drinking is the most prevalent form of alcohol abuse (World Health Organization, 2011; National Center for Chronic Disease Prevention and Health Promotion, 2012) and, at least in the United States, more than half of the total binge drinks are consumed by those aged 35 years or older (Kanny et al., 2018). Very concerning, a published survey of individual drinking patterns during the early part of the coronavirus (COVID-19) pandemic indicated a 41% increase in heavy drinking reported by mature adult women, particularly those between the ages of 30 and 59, over their pre-pandemic baseline (Pollard et al., 2020). Such marked increases in heavy alcohol consumption by mature adult women are particularly concerning in light of their reported greater vulnerability to alcohol-induced cancer, heart, and liver disease (c.f., Agabio et al., 2017), and greater propensity to develop AD and related dementias (e.g., Hebert et al., 2013; Ferretti et al., 2018). Thus, is it imperative that we investigate how biological sex interacts with a history of heavy drinking during mature adulthood to impact cognitive function and contribute to AD-AUD comorbidity?

A large number of confounding variables render it near-impossible to disentangle cause-effect relations between brain function and behavior in humans with alcohol abuse or AUD (Volkow and Li, 2005; Nunes et al., 2019). In this regard, animal models of heavy alcohol drinking or binge drinking provide a powerful tool with which to study simultaneously the pathobiology of excessive drinking and its behavioral correlates, including effects on cognition. One strategy to address alcohol’s impact on AD-like symptoms is to assay the effects of alcohol in transgenic mouse models of AD that develop cognitive decline with aging, in concert with the manifestation of biomarkers of AD (e.g., accumulation of phosphorylated tau, Aβ1–40, Aβ1–42, neuritic plaque formation, etc.; e.g., Webster et al., 2014; Hu et al., 2015; Jankowsky and Zheng, 2017). For example, the APP/PS45 murine model of AD exhibits cognitive deficits, as well as increased expression of APP, BACE1, Aβ1–40, and Aβ1–42, and increased neuritic plaque formation within the hippocampus, following experimenter-administered, binge-like, alcohol exposure during the adolescent/early adult period of development (Ledesma et al., 2020). In the 3XTg-AD mouse model, a 4-month history of voluntary alcohol consumption under continuous-access procedures during adulthood (10 weeks of age) results in a number of AD-like behavioral pathologies, including impaired spatial memory, sensorimotor gating, and exacerbated conditioned fear, which were associated with an upregulation in biomarkers of AD pathology (Hoffman et al., 2019). The limited data to date support the notion that chronic (i.e., at least 1 month) alcohol exposure can exacerbate the onset or severity of AD-like behavioral and neurological symptoms in transgenic models of AD vulnerability.

The issue of whether or not a history of heavy drinking can impact the onset and/or severity of AD-like behavioral and brain pathology in wild-type (i.e., genetically non-manipulated) rodents has received even less experimental attention. However, there is consensus in the aging literature that the best animal model for both the normal and pathological aging process is the aging animal itself (Yamada and Nabeshima, 2000; Scharl et al., 2005). Here in, we tested the hypothesis that a history of binge drinking during mature adulthood [i.e., pre-middle-age (between 6 and 9 months old); Flurkey and Harrison, 2007] impairs spatial and working memory to a greater extent in female vs. male C57BL/6J (B6J) mice and that the severity of this impairment would be comparable to that of an old (18 month-old) animal. The B6J strain was selected for study for a number of reasons. First, this strain exhibits a high alcohol-preferring and consuming phenotype (e.g., McClearn and Rodgers, 1959; Belknap et al., 1993; Wahlsten et al., 2006). Second, both adolescent and adult C57BL/6J mice reliably binge-drink alcohol over the course of 1 month under different limited-access procedures (Cozzoli et al., 2009; Lee et al., 2015), with female mice binge drinking larger amounts of alcohol than males (e.g., Finn et al., 2005; Rhodes et al., 2005; Szumlinski et al., 2019; Jimenez Chavez et al., 2020). Third, a sex difference is reported in age-related cognitive decline in alcohol-naïve B6J mice (Benice et al., 2006), as well as the related C57BL/6NIA strain (Frick et al., 2000). Fourth, binge drinking under conventional Drinking-in-the-Dark (DID) procedures during earlier adulthood (2–3 months of age) induces microglia activation within the hippocampus of C57BL/6N mice (Grifasi et al., 2019), whereas binge drinking under modified DID procedures by adolescent B6 mice elicits cognitive impairment in adulthood (Van Hees et al., 2022). Finally, voluntary, non-dependence, drinking alters a number of AD-related genes within the brains of both adolescent and adult B6J mice (Salling et al., 2016; Hoffman et al., 2019).

Based on these findings, we hypothesized first that mature adult female B6J mice would binge-drink a larger amount of alcohol than males. Second, we hypothesized that female B6J mice would exhibit poorer signs of cognitive performance than males, regardless of their alcohol drinking history as indicated by one or more of the following outcomes: (1) a longer latency to locate the hidden platform(s) during the acquisition phases of both Morris water maze and radial arm maze testing; (2) less time spent in the quadrant of the Morris water maze during a memory probe test; (3) poorer reversal learning when the platform location was changed in the Morris water maze; (4) more reference and working memory errors during the acquisition of the radial arm maze; and (5) a greater reliance on non-working memory strategies to navigate the radial arm maze, as index by chaining behavior (see Section “Methods”). Third, we hypothesized that a history of binge drinking would augment the aforementioned signs of cognitive impairment in both male and female mice, but that the severity of the impairment would be more robust or comprehensive in female than male subjects.

In all, three studies were conducted. The first experiment was a pilot study originally designed to assay the behavioral effects of binge drinking during middle-age (i.e., 12-month-old mice; Flurkey and Harrison, 2007). However, institutional research shut-down due to the COVID-19 pandemic, coupled with uncertainty over the timing of research re-opening, prompted us to assay the available isogenic and congenic B6J mice in our colony at an earlier age than originally intended. As such, Experiment 1 was conducted in a genetically heterogeneous cohort of mice, that ranged in age from 5 to 9 months, with varying, but brief, prior experiential histories (see Table 1 for details). To address interpretational confounds associated with the varied experiential and/or genetic backgrounds of the mice in Experiment 1, Experiment 2 employed solely isogenic B6J male and female mice between 6 and 6.5 months of age, derived from the Jackson Laboratory. Experiment 3 served to extend the results from Experiments 1 and 2 and did so in two ways. First, it included both mature adult and oldB6J mice (18 months of age; Flurkey and Harrison, 2007) to: (1) experimentally address the influence of age on binge drinking, which was indicated by the results of Experiment 1; (2) confirm that a binge drinking history elicits cognitive abnormalities in mature adult mice comparable to those of a more aged animal; and (3) determine if age-related cognitive decline is exacerbated by binge drinking and sex differences therein.

**Table 1:** Detailed description of the composition of the subjects employed in Experiment 1.

			**Water**		**DID**	
**Age (mo.)**	**Source**	**Prior history**	**Male**	**Female**	**Male**	**Female**
**5**	JAX	naïve; left undisturbed under reverse cycle from 2.5 to 5 months of age	4 B6J	1 B6J	4 B6J	2 B6J
**7**	UCSB	(1) @ 2-2.5 months of age: 2, 60-min, locomotor habituation sessions + 15 mg/kg cocaine (1 h activity session) (2) left undisturbed under reverse cycle for ~4.5 months	2 Hn1 WT	4 Hn1 WT	4 Hn1 WT	
**8**	UTSW	(1) shipped to UCSB @ 2–3 months of age + 7 week quarantine (2) housed under reverse cycle for 2 weeks (3) 3 days of 20% sucrose drinking (1 h/day) with 30 mg/kg cocaine injection administered after session 3 + final sucrose drinking test (1 h) on day 4 (5) undisturbed for ~3 months	1 H2 WT	2 H2 WT	3 H2 WT	5 H2 WT
	UCSB	(1) @ 3–3.5 months of age: 2, 60-min, locomotor habituation sessions + 15 mg/kg cocaine (1 h activity session) (2) left undisturbed under reverse cycle for ~4.5 months	2 Hn1 WT	1 Hn1 WT		
**9**	UCSB	(1) @ 4–4.25 months of age: 2, 60-min, locomotor habituation sessions + 15 mg/kg cocaine (1 h activity session) (2) left undisturbed under reverse cycle for ~4.5 months		4 Hn1 WT		
**Totals**			9 males	12 females	11 males	7 females

*Note that isogenic C57BL/6J (B6J) males and females were experimentally-naïve. Wild-type (WT) mice on a congenic B6J background from our colony of *Hnrnph1*+/+ and +/– mice (Hn1) and from our colony of *Homer2^S117/S216^*and *Homer2^A117/A216^* (H2) were injected once with 15 and 30 mg/kg cocaine, respectively, 3–4 months prior to alcohol drinking procedures*.

A history of binge drinking elicits a negative affective state during early alcohol withdrawal in both male and female young adult mice (i.e., mice aged 2–3 months; Lee et al., 2015; Szumlinski et al., 2019; Jimenez Chavez et al., 2020); however, the affective consequences of binge drinking have not been explored in older animals. To address the potential contribution of withdrawal-induced anxiety to the severe impairment in visually-cued navigation exhibited by the binge drinking mice of both Experiments 1 and 2, all mice in Experiment 3 were first screened for a negative affective state, the day prior to commencing cognitive testing procedures. Through this series of studies, we show that a history of binge drinking by both mature adult and old mice induces a robust negative affective state that is relatively sex-independent. Moreover, we show that binge drinking during mature adulthood accelerates cognitive impairment in both male and female subjects, with females exhibiting more signs of impairment than males. In contrast, binge drinking by old mice does not worsen their cognitive performance, indicating that the ability to detect the cognitive-impairing effects of binge drinking is age-dependent.

## Methods

### Subjects

#### Experiment 1

As stated in the Introduction, institutional research shutdown at UCSB due to the COVID-19 pandemic prompted us to commence a study into the behavioral effects of binge drinking in later life using the available colonies of mice housed in our vivarium at the time of the study. These mice varied in terms of their age of binge drinking onset (5–9 months of age), as well as their genetic and research experiential backgrounds. As summarized in Table 1, a proportion of the Experiment 1 mice were on an isogenic B6J background and were purchased from Jackson Laboratories (Sacramento, CA). The mice arrived at our facility on ~PND 65 and then were left undisturbed in the home cage, under a reversed light cycle (lights off at 1100 h) until drinking procedures commenced (i.e., were experimentally naïve).

Some of the other mice employed in Experiment 1 were on a congenic B6J background and included wild-type (WT) mice from our colony of *Hnrnph1+/+* and *+/–* animals maintained at UCSB (see Ruan et al., 2020). These mice were weaned on PND21 and housed in groups of 2–4 with same-sex littermates under a regular light cycle (lights on at 0700 h) for 2–4 months at which time, they underwent locomotor testing. For this, mice were habituated to Plexiglas locomotor activity chambers (40 cm × 40 cm × 40 cm) for 60 min/day across 2 days and then were tested for their locomotor response to an acute, intraperitoneal, injection of 15 mg/kg cocaine over a 1-h session. The next day, the WT *Hnrnph1+/+* (Hn1 WT) mice from this cocaine study were housed under the reverse light-dark cycle for approximately 4.5 months until drinking commenced (see Table 1).

Other congenic B6J mice were derived from our colony of transgenic *Homer2^S11A7/S216A^* mice, generated by the laboratory of Dr. Kimberly Huber (UT Southwestern). These mice arrived at UCSB on ~PND40–50, were housed in same-sex groups of 4, and quarantined for an additional 7 weeks, prior to housing in the main vivarium under the same reverse light-dark cycle as the other Experiment 1 mice. Following 2 weeks of acclimation to the reverse cycle, the *Homer2* transgenic mice (~PND 120) were subjected to a cocaine-induced taste aversion experiment. For this, mice were individually housed in standard mouse cages with a wire top situated on a free-standing rack in the colony room at 2 h into the dark phase of the cycle (~1300 h). One hour later (~1400 h), mice were presented with a single sipper tube containing 20% sucrose (w/v) and allowed to drink for 1 h. This sucrose drinking procedure continued for 3 days. Immediately following the third sucrose drinking session, mice were injected, intraperitoneally, with 30 mg/kg cocaine and returned to their home cage. The next day, the sucrose drinking procedure was repeated to examine for taste aversion. Upon the completion of aversion testing, the mice were returned to their home cage and left undisturbed for ~3 months until alcohol drinking commenced. For the congenic B6J mice, only WT mice were included in this study to avoid potential interactions between the mutation and binge drinking (e.g., Fultz et al., 2021).

All of the mice in Experiment 1 were run simultaneously as one cohort of animals. One DID male was euthanized following Morris water maze testing due to a severe bite wound that did not respond to treatment while one Water male was found inexplicably dead in its home cage during radial arm testing. Thus, the final sample sizes employed in Experiment 1 were: Female-Water = 11, Female DID = 7, Male-Water = 11 for Morris water maze and *n* = 10 for radial arm maze, and Male-DID = 11 for both drinking and Morris water maze, but *n* = 10 for radial arm maze.

#### Experiment 2

The data from Experiment 1 indicated some effects of prior binge drinking history on our cognitive measures and the results of the analyses of covariance (ANCOVAs) indicated that genetic/experimental background and/or age significantly adjusted the data for both binge drinking and the majority of our cognitive outcomes. Thus, to control for both variables, Experiment 2 was conducted using exclusively 6 month-old, male and female, mice on an isogenic B6J genetic background, and all mice were purchased from the Jackson Laboratory. Mice were housed in same-sex and same experimental condition groups of 3–4 under standard ventilated caging and reverse cycle housing conditions (lights off at 1100 h). The first cohort of B6J mice employed in Experiment 2 consisted of 24 male (12 Water and 12 DID) and 13 female B6J mice (7 Water and 6 DID). These mice arrived at our facility on PND22 and were housed under our reverse light-dark cycle until they commenced drinking procedures at 6 months of age—a time corresponding to late maturity in the B6J strain (Flurkey and Harrison, 2007). To increase the sample sizes for the females, an additional 10 female 4.5-month-old B6J mice (5 Water and 5 DID) were purchased from the Jackson Laboratory and were housed for 1.5 months under the reverse light-dark cycle prior to undergoing drinking procedures. This second cohort of female mice underwent binge drinking procedures approximately 1 month following that of the larger, mixed-sex, cohort. The cognitive behavioral data for one female DID mouse from the first cohort (mouse 18) was discarded based on an outlier analysis. Thus, the final sample sizes employed in Experiment 2 were: Female-Water = 12, Female-DID = 10, Male-Water = 12, and Male-DID = 12.

#### Experiment 3

The ANCOVA results from Experiment 1 also indicated that age significantly adjusted the data for both binge drinking and the majority of our cognitive outcomes (see Section “Results”). Thus, Experiment 3 was conducted to directly examine for age differences in the effects of binge drinking on cognition. For Experiment 3, B6J mice arrived simultaneously at our facility at either 5 or 17 months of age and were housed for 1 month under our reverse light cycle prior to undergoing drinking procedures. The 6-month-old mice in Experiment 3 provided an unadulterated replicate of Experiment 2, while the 18-month-old mice served as a comparator for the severity of alcohol-induced cognitive impairment in the 6-month-old animals and to examine directly for age by sex interactions in the effects of binge drinking on our cognitive measures. At the start of Experiment 3, the samples sizes were 12 for each group, with the exception of the 18-month-old male Water control group, which had a sample size of 14 as two additional 18-month-old males arrived with the shipment and were placed in the control group to increase the statistical power of the study. One female 18-month-old water control was euthanized prior to behavioral testing for remaining underweight (~19 g) throughout the drinking phase of the study.

For all experiments, the mice were housed in standard polycarbonate cages on a ventilated rack in a climate- and humidity-controlled holding room. Food and water were available *ad libitum* except during behavioral testing. All the cages were lined with sawdust bedding, nesting materials, and a plastic enrichment device in accordance with vivarium protocols. All experimental procedures were in compliance with The Guide for the Care and Use of Laboratory Animals (National Research Council, 2011) and approved by the Institutional Animal Care and Use Committee of the University of California, Santa Barbara.

### Drinking-in-the-Dark (DID) Procedures

Approximately half of the mice in each cohort were subjected to 30 consecutive days of binge drinking using a multi-bottle-choice DID procedure that involved concurrent access to unadulterated 10, 20, and 40% (v/v) ethanol in tap water (e.g., Cozzoli et al., 2012; Lee et al., 2016). At 2 h after lights out (i.e., 1300 h), alcohol-drinking (DID) animals were transferred to individual drinking cages that were lined with sawdust bedding and topped with a wire lid, situated on a free-standing rack within the holding room. Mice were allowed to habituate to the drinking cage for 1 h, at which time, the three sipper tubes containing the alcohol solutions were placed on the drinking cage, with the location of the sipper tubes randomized daily. Animals were allowed to drink for 2 h (1400–1600 h). At 1600 h, the sipper tubes were removed from the drinking cages and the DID mice were then transferred back into their home cages. As a result of other ongoing drinking studies, there was insufficient space in the holding room to individually house water control mice (Water) from Experiment 1 and the large size of the Experiment 3 study precluded singly-housing the Water mice involved in this study. Thus, the Water mice in Experiments 1 and 3 were handled daily by being placed, with their cage mates, into a novel drinking cage on the same free-standing rack as the DID mice and were presented with a single sipper tube containing water, as conducted in prior work (e.g., Lee et al., 2018b; Szumlinski et al., 2019; Jimenez Chavez et al., 2020). In Experiment 2, both the DID and Water animals were housed in individual drinking cages during the 3 h period, with the Water mice presented with one sipper tube filled with water during the 2 h drinking session (e.g., Cozzoli et al., 2012; Lee et al., 2016). As mice are not fluid-deprived under DID procedures, it has been our experience over the past 15 years of conducting such studies that Water controls consume very little water during a 2-h drinking session and that the total volume consumed is very low (~10%–15% the volume of alcohol consumed) regardless of the number of mice in the drinking cage or the number of water-containing sipper tubes presented. Thus, over the years, we have simplified our water control procedures to facilitate the testing of large cohorts of mice to include measuring only the volume of consumed from alcohol-containing sipper tubes.

At the end of each 2 h drinking session, the mice were returned to their home cages and placed back on the ventilated rack. In all experiments, the alcohol-containing sipper tubes were weight prior to, and immediately following, each 2 h drinking session to determine the volume consumed. The alcohol/water in the bottles was refreshed and all of the mice were weighed every 3–4 days during the drinking procedures. The recorded body weights of the mice were used to calculate alcohol intake.

### Blood Ethanol Concentration

On the 20th drinking day in Experiments 1 and 2 and on the 30th (last) drinking day in Experiment 3, submandibular blood samples were collected from the alcohol-drinking mice only, immediately after the 2 h alcohol-drinking period. The latter time-point in Experiment 3 merely reflected the availability of trained laboratory personnel to conduct the blood sampling procedures. Samples were stored at −20°C until processing. As conducted in recent reports (e.g., Fultz and Szumlinski, 2018; Jimenez Chavez et al., 2020, 2021), headspace gas chromatography was employed to analyze blood ethanol concentrations (BECs) and, with the exception of the male isogenic B6J mice, BECs were determined within 7–10 days of sample collection. BECs were determined using a Shimadzu GC-2014 gas chromatography system (Shimadzu, Columbia, MD), and the data was determined *via* the GC Solutions 2.10.00 software. Samples were diluted at 1:9 with non-bacteriostatic saline (50 μl of the sample). Chloroform was used as the pre-solvents and the determination of ethanol from each sample was derived using the standard curve equation determined prior to analyses of the blood samples. A new standard curve was formulated for each cohort of blood samples to ensure maximal accuracy. After the ethanol peak area was determined, the peak area was used to determine the ethanol concentration and subsequently the percent of ethanol in the blood.

### Morris Water Maze

**Figure 1:**
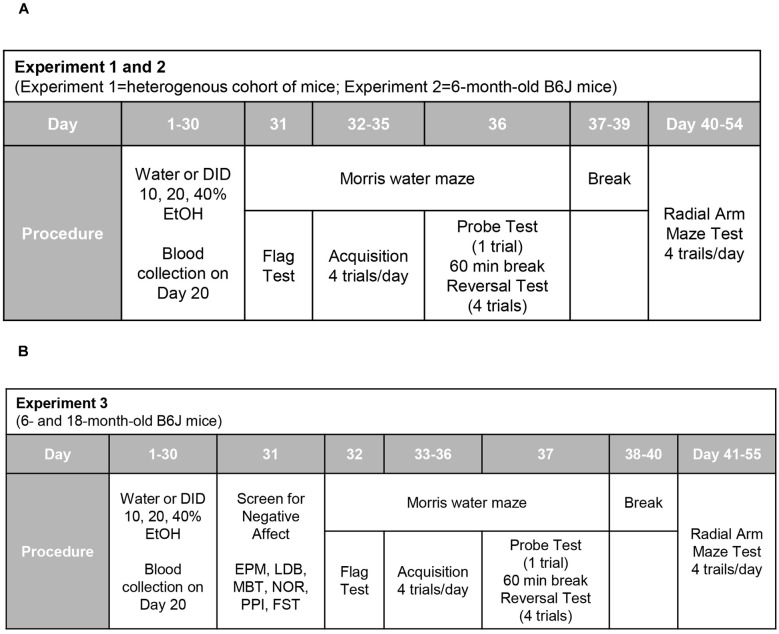
Procedural time-lines of Experiments 1, 2, and 3. **(A)** In both Experiments 1 and 2, mice underwent a month-long period of binge drinking, followed first by training and testing in a Morris water maze and then, by training in a water version of a radial arm maze. **(B)** The procedural time-line for Experiment 3 was similar to that of the other two experiments, with the exception that mice underwent a 1-day behavioral test battery for negative affect prior to Morris water and radial arm maze testing.

The day following drinking (for Experiments 1 and 2; Figure 1A), or the day following testing for negative effects (Experiment 3; Figure 1B), all mice were assayed for spatial learning and memory using Morris water maze procedures akin to those published previously by our laboratory (e.g., Lominac et al., 2005; Ary et al., 2013; Datko et al., 2017). The maze consisted of a stainless-steel circular tank (200 cm in diameter, 60 cm in height; filled with room temperature water to a depth of 40 cm), with salient intra-maze cues located on all four sides of the tank. To ensure equivalent visual processing in all mice at the outset of each experiment, Morris maze testing commenced with a “flag test”, in which the clear platform was placed in the tank in the NW quadrant with a patterned flag attached that extended 6 inches above the water. While the majority of mice located the flagged platform during the 2-min period, some mice required 1–2 additional 2-min training sessions prior to locating the flagged platform. Over the course of the next 4 days, the clear platform remained in a fixed location in the NE quadrant (i.e., a quadrant distinct from that employed in the flag test). Each day, mice were trained four times a day (once at each compass point) to locate the hidden platform. During each trial, mice were randomly placed in the pool at one of the four compass points and swimming was recorded digitally by a video camera mounted on the ceiling directly above the pool (ANY-Maze, Stoelting). Training sessions were 2-min in duration and mice were tested in series at each compass release point. Mice unable to locate the platform during the allotted time were guided to the platform using forceps, where they remained for 30 s. At 24 h after the last training trial, a 2-min memory probe test was performed in which the platform was removed from the pool and the amount of time taken by the mouse to swim toward the former platform location and the time spent swimming in the NE quadrant that formerly contained the platform was recorded (Lominac et al., 2005; Ary et al., 2013; Datko et al., 2017). At least 1 h following the Probe test, a reversal training session was conducted in which the platform (unflagged) was situated in the SW quadrant (i.e., the quadrant opposite to that employed during the training phase of the experiment). Again, mice were trained to locate the platform over four, 2-min, sessions (one training trial for each compass point) to locate the repositioned platform. The timeline for the different procedures employed during Morris water maze testing is presented in Figure 1.

### Water Version of the Radial Arm Maze

Following a 2–3 day break from Morris maze testing (see Figure 1), working and reference memory were determined using a water version of the radial arm maze with procedures similar to those employed in our prior studies (Lominac et al., 2005; Szumlinski et al., 2005). The maze consisted of eight arms with clear, hidden, escape platforms at the ends of four of the arms. The start arm was the same for all the mice and remained constant throughout. Each mouse was assigned different platform locations that remained fixed throughout the experiment and the baited arms were semi-randomly assigned across subjects. A subject had 120 s to locate a platform. If the mouse was unsuccessful at locating a platform in the allotted time, it was guided to the nearest available platform using forceps. Once a platform was found, the animal remained on it for 15 s, and was then returned to an empty, heated, holding cage for 30 s. During that time, the located platform was removed from the maze. The animal was then placed back into the start arm and allowed to locate another platform. Each day, this sequence of events repeated until the mouse located all four platforms. Thus, each mouse underwent four trials per day, with the working memory system taxed increasingly with each trial. This version is similar to the land version of the radial-arm maze in that animals have to avoid arms that never contained a reinforcer (reference memory) and enter only once into arms that contained a reinforcer (working memory). Day 1 was considered a training session because the animal had no previous experience in the maze. Days 2–14 were testing sessions and errors were quantified for each day using the orthogonal measures of working and reference memory errors (Jarrard et al., 1984), as conducted previously by our group (Lominac et al., 2005; Szumlinski et al., 2005) and others (e.g., Bimonte et al., 2000). Working memory correct errors were the number of first and repeat entries into any arm from which a platform had been removed during that session. Reference memory errors were the number of first entries into any arm that never contained a platform. Working memory incorrect errors were the number of repeat entries into an arm that never contained a platform in the past (thus, repeat entries into a reference memory arm). In addition to these measures of working and reference memory, “chaining” behavior was also recorded. Chaining refers to the number of consecutive entries into two adjacent arms and represents an alternate strategy to maze navigation that is often exhibited by cognitively impaired subjects (e.g., Szumlinski et al., 2005).

### Behavioral Test Battery for Negative Effect

To address the possibility that the anomalies in visually-cued spatial navigation exhibited by the DID mice in Experiments 1 and 2 (see Section “Results”) might reflect alcohol withdrawal-induced negative effect, the mice in Experiment 3 first underwent a behavioral test battery for negative affect prior to cognitive testing (see Figure 1B). As in our prior studies of adolescent and young adult (2 month-old) mice (e.g., Lee et al., 2015; Szumlinski et al., 2019; Jimenez Chavez et al., 2020), this behavioral test battery was conducted 24 h following the last drinking session and consisted of several paradigms that were administered in a random fashion, including light-dark shuttle-box, elevated plus-maze, marble-burying, novel object reactivity test, and acoustic startle. At the end of each of these procedures, mice were returned to their home cages, which were situated on a cart in the procedural room. As in our aforementioned prior studies of adolescent and younger adult mice, screening for negative effect concluded with a forced swim test, following which animals were allowed to rest and dry completely in a holding cage prior to being returned to their home cages. The behavioral testing equipment was cleaned in-between each use with Rescue Disinfectant Veterinary Wipes (Virox Animal Health, Oakville, ON, Canada). The details of each specific assay are provided below and are similar to those described in the reports listed above. Based on the results of a recent study (Jimenez Chavez et al., 2020), males and females were tested for negative effect on separate days to minimize any pheromonal influences on affective behavior.

#### Novel Object Reactivity Test

To test reactivity to a novel object as an index of neophobia-related anxiety (Misslin and Ropartz, 1981; Dulawa et al., 1999), mice were tested in a Plexiglas activity chamber (46 cm long × 42 cm wide × 40 cm high), in the center of which was placed a novel, inedible, object (candlestick holder; measuring approximately 6 cm in diameter × 12 cm high). The animals were allowed to explore the chamber over a 2-min trial during which the number of contacts, total time spent in contact with the novel object, and fecal count were recorded by a trained observer who was blind to the drinking condition of the animals.

#### Acoustic Startle and Pre-pulse Inhibition of Acoustic Startle

The apparatus and procedures employed to assay the magnitude of acoustic startle and prepulse inhibition of acoustic startle were similar to those described previously by our group (e.g., Lominac et al., 2005; Szumlinski et al., 2005; Datko et al., 2017). Six different trial types were presented: startle pulse (st110, 110 dB/40 ms), low prepulse stimulus given alone (st74, 74 dB/20 ms), high prepulse stimulus given alone (st90, 90 dB/20 ms), st74 or st90 given 100 ms before the onset of the startle pulse (pp74 and pp90, respectively) and no acoustic stimulus (i.e., only background noise was presented; st0). St100, st0, pp74, and pp90 trials were applied 10 times, st74 and st90 trials were applied five times, and all trials were given in random order. The average intertrial interval was 15 s (10–20 s), and the background noise of each chamber was 70 dB. The data for startle amplitude were averaged across each of the stimulus trial types for statistical analyses of startle magnitude. The percent inhibition of the 110 dB startle by the 74- and 90-dB prepulse intensities was also calculated for each animal.

#### Light–Dark Shuttle-Box

The light–dark shuttle-box was also used to measure photophobia, with decreased activity on the light-side interpreted as reflecting an anxiety-like phenotype (Crawley, 1985; Gallo et al., 2004). Animals were placed into a polycarbonate box (46 cm long × 22 cm wide× 24 cm high) that was divided into two environments, one side was white with a clear lid and the other side was black with a black lid (respectively, light vs. dark side) that were accessible through a central divider with an opening. Testing commenced by placing the mice in the dark environment. The latency to enter the light side, total time spent in the light side, and the total number of light entries were recorded over a 5-min period by trained experimenters blind to the prior drinking histories of the mice.

#### Marble-Burying Test

The marble-burying test is particularly sensitive to the anxiogenic effects of alcohol withdrawal, based on our prior work with adolescent and young adult (i.e., 2–3 month-old) mice (e.g., Lee et al., 2015, 2016, 2017a, b, 2018a, b; Szumlinski et al., 2019; Jimenez Chavez et al., 2020). For this assay, mice were placed in a polycarbonate cage (12 cm × 8 cm × 6 cm), with 5-cm deep sawdust bedding on top of which 25 black marbles were arranged equidistantly. Mice were left undisturbed for a period of 20 min at which time, the percent of marbles buried (i.e., 75% covered by bedding) was determined by an experimenter who was blind to the drinking history of the mice.

#### Elevated Plus Maze

The elevated plus maze is a well-established paradigm in which to measure anxiety-like behavior in laboratory animals, with high predictive validity for anxiolytic drugs (Karl et al., 2003; Walf and Frye, 2007). Animals were placed on the center intersection of a 4-arm radial plus maze with two white open arms and two black walled arms 24 cm high. Each arm measured 123 cm long × 5 cm wide. Latency to first open-arm entry, number of open-arm entries, and total time spent in an open arm were monitored for the 2-min trial by a trained observer who was blind to the drinking history of the mice. The amount of time spent in the open arm was also used to assess anxiety-like behavior.

#### Forced Swim Test

The forced swim test is a commonly employed assay for the reversal of passive coping behavior by anti-depressant treatments (Porsolt et al., 2001). Excessive swimming behavior in this assay can be reversed by pretreatment with anxiolytic medications and thus, has been used by our group as an additional measure of anxiety-like behavior during alcohol withdrawal (Lee et al., 2015, 2016, 2017a, 2018a, b; Szumlinski et al., 2019; Jimenez Chavez et al., 2020). In our paradigm, an 11-cm diameter cylindrical glass container is filled with room temperature water, and animals are tested over a 6-min period during which AnyMaze^TM^ tracking software determined the latency to the first immobile episode, total time spent immobile, and the number of immobile episodes. Immobility is defined as the lack of vertical or horizontal displacement of the animal’s center of gravity for at least 5-s. Upon the conclusion of this assay, animals were allowed to dry prior to being returned to their home cage and the holding room.

### Statistical Analysis

As the mice employed in Experiment 1 were heterogeneous with respect to both their age at drinking/testing and their genetic/experimental background, the data for Experiment 1 were analyzed using an ANCOVA adjusting for both age and the genetic/experimental background as co-variates, with the between-subjects factors of History (Water vs. DID) and Sex. As one or both factors were found to significantly adjust the means for the vast majority of our dependent variables (see Section “Results”), all data from Experiment 1 are presented as adjusted means ± SEMs. A subset of the female mice in Experiment 2 (*n* = 5/Drinking History) were housed for only 1.5 months under a reverse light-dark cycle, while all of the male mice and the other fraction of female mice were housed under a reverse light-cycle since weaning (see Section “Subjects”). Thus, the data for Experiment 2 were also analyzed using an ANCOVA, adjusting for light-cycle as a covariate. The results of the statistical analyses indicated no adjustment of the means by the light-cycle covariate; thus, all data from Experiment 2, as well as the data from Experiment 3, are presented as the actual means ± SEMs. As there were no overt confounding variables in Experiment 3, the data were analyzed using multi-variate ANOVAs with the between-subjects factors of history and sex, with age (6 vs. 18 months old) also included as a between-subjects factor. Depending upon the paradigm, the within-subjects factors of day (for drinking), session (for maze acquisition), trial (for reversal learning), or pre-pulse (for PPI) were also included in the analyses. Significant interactions were deconstructed along the relevant factors, followed by *post-hoc* tests for main effects (when multiple comparisons were involved) or *t*-tests (when <3 comparisons were involved). Statistical outliers were identified for each test using the _1.5_IQR rule and excluded from the analysis. Alpha was set at 0.05 for all analyses.

## Results

### Experiment 1. Heterogenous Mouse Population of Congenic and Isogenic B6J Mice

Statistical analyses of the data by ANCOVA, adjusting for the difference in age of testing and the genetic/experimental background of the mice (see Table 1) revealed either significant effects of one or both of these covariates for the majority of our measures (i.e., *p’s* < 0.05), or strong statistical trends towards a covariate effect (i.e., *p’s* = 0.06–0.08). Thus, the data from Experiment 1 are presented as the adjusted means ± SEMs and all statistical results reflect the analysis of these adjusted means.

#### Alcohol Intake and BECs

**Figure 2:**
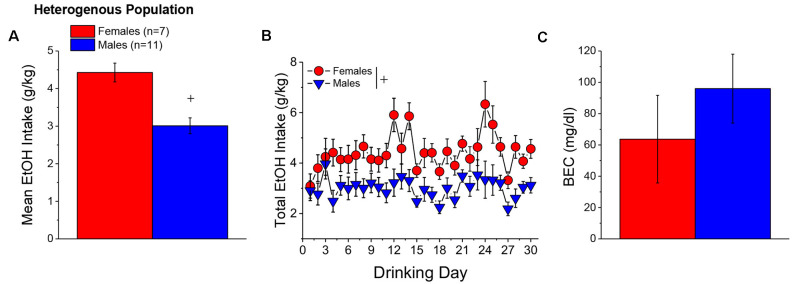
Binge drinking during later maturity (6–9 months of age) in a heterogenous cohort of mice. **(A)** On average, females in Experiment 1 consumed more alcohol than males over the 30-day course of binge drinking. **(B)** Comparison of the time-courses of binge drinking between Experiment 1 male and female mice. **(C)** Despite the sex difference in binge drinking, BECs were comparable between Experiment 1 male and female mice. The data represent the means ± SEMs, adjusted for the covariates of age and background, of the number of mice indicated in parentheses in panel **(A)**. ^+^*p* < 0.05 Males vs. Females (sex difference).

When controlling for both age and genetic background, the female mice in Experiment 1 (*n* = 7) consumed more alcohol, on average, over the course of binge drinking than males (*n* = 11; Figure 2A) [Sex effect: *F*_(1, 14)_ = 16.39, *p* = 0.001], with no sex difference detected for the time-course of drinking over the 30-day period (Figure 2B; Day effect and Day × Sex: *p’s* > 0.50). Adjusting for age and genetic background, male (*n* = 11) and female (*n* = 7) mice consumed on average 4.63 ± 0.35 g/kg and 3.10 ± 0.28 g/kg alcohol, respectively, on the day of blood sampling and the intake on this day was significantly higher in females than in males [Sex effect: *F*_(1, 17)_ = 10.67, *p* = 0.006]. The adjusted data for the BECs attained on that day of blood sampling were quite variable, with the adjusted mean BEC of males exceeding the 80 mg/dl criterion for binge drinking, while that of females fell within error of that criterion (Figure 2C). Despite the sex difference in binge drinking on the day of blood sampling, we detected no sex difference in BECs (Figure 2C; Sex effect: *p* = 0.39). These data indicate that mice aged 5–8 months engage in binge drinking under our 2-h DID procedures.

#### Morris Maze

##### Flag Test

**Figure 3:**
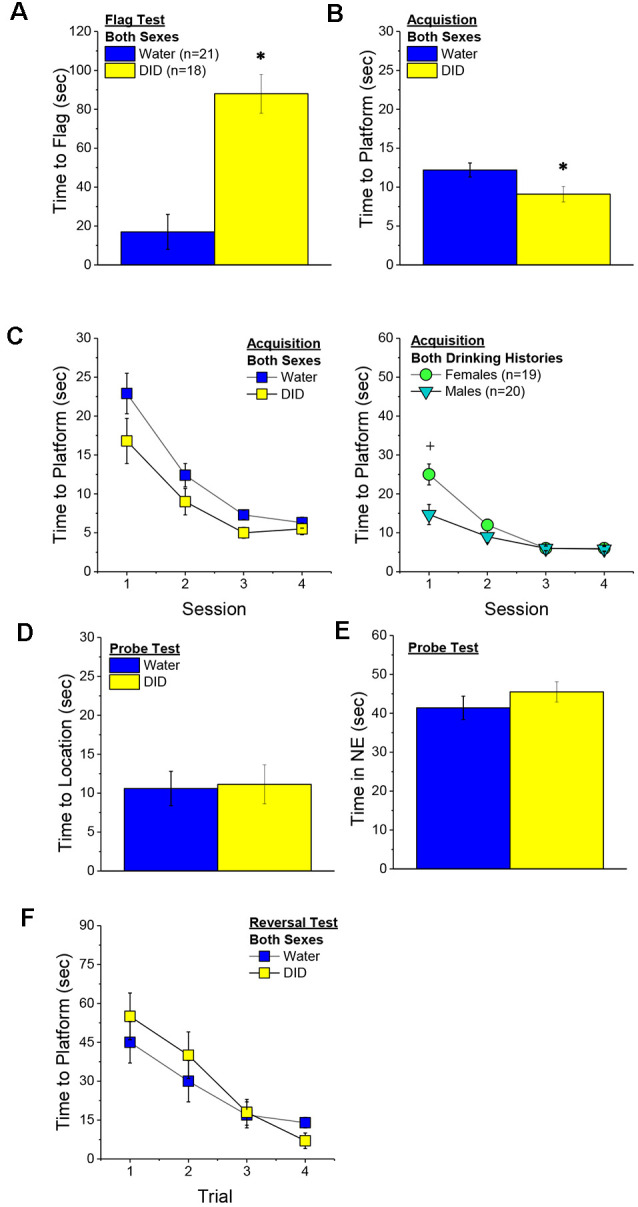
Effects of binge drinking during later maturity (6–9 months of age) on Morris water maze performance in a heterogenous cohort of mice. Relative to water-drinking controls (Water), the binge drinking mice (DID) in Experiment 1 exhibited a longer latency to locate a visible, flagged, platform **(A)**, but an average shorter latency to locate the hidden platform during the acquisition phase of the study **(B)**. **(C)** Comparison of the time-course of spatial learning in the Morris water maze between DID and Water animals collapsed across sex **(left)** and between males and females, collapsed across drinking history **(right)**. On the probe test, no group differences were detected for the latency to first enter the former platform location **(D)** or the total time spent in the NE quadrant that formerly contained the platform **(E)**. **(F)** No group differences were detected for reversal learning then the hidden platform is moved to a new location in the maze. The data represent the means ± SEMs, adjusted for the covariates of age and background, of the number of mice indicated in parentheses. *p < 0.05 DID vs. Water; ^+^*p* < 0.05 Females vs. Males.

After controlling for both age and genetic background, we detected a sex-independent increase in the latency of DID mice to locate a visually-cued platform during the flag test, compared to Water controls (Figure 3A) [Drinking History effect: *F*_(1, 38)_ = 24.93, *p* < 0.0001; Sex × Drinking History, *p* = 0.56]. In fact, half of the 18 DID mice did not locate the flagged platform during the initial test, while only one of the 21 Water controls failed initially to locate the platform (*λ*^2^ = 6.19, *p* = 0.01). The one Water mouse and seven of the nine DID mice were able to locate the platform on the 2nd trial, while the two remaining DID mice required a third trial before completing the task. These data indicate that a history of binge drinking during mature adulthood disrupts visually guided escape behavior. An analysis of the distance traveled during the flag test indicated that DID mice swam approximately five times a greater distance than Water mice during this test [adjusted means, Water: 8.75 ± 5.87 m vs. DID: 49.82 ± 6.64 m; History effect: *F*_(1, 38)_ = 18.65, *p* < 0.0001; Sex effect and interaction, *p’s* > 0.50], the longer latency or inability to locate the visible platform does not reflect a motor impairment or a higher level of floating/passive coping behavior in the DID mice.

##### Maze Training

When controlling for age and genetic background, we detected an overall effect of prior binge drinking history on the latency of mature adult mice to locate the hidden platform across the 4 days of training [History effect: *F*_(1, 33)_ = 4.65, *p* = 0.04]. However, contrary to our findings for the Flag Test, DID mice were slightly, but significantly, quicker to locate the hidden platform during maze acquisition than Water controls (Figure 3B). The ANCOVA detected no significant effects of binge drinking history on the time-course of learning (Figure 3C; History × Session interactions, *p’s* > 0.13). However, we did detect a sex difference in learning as indicated by a significant Sex × Session interaction [*F*_(3, 99)_ = 5.25, *p* = 0.002]. As illustrated in Figure 3D, this interaction reflected a longer latency for female vs. male mice to locate the hidden platform on the first training session only [one-way ANCOVAs across Sex: for Session 1, *F*_(1, 38)_ = 8.46, *p* = 0.006; for Sessions 2–4, all *p’s* > 0.08]. These data for the Morris water maze acquisition indicate that mature adult female mice exhibit initial deficits in spatial learning, but that binge drinking during mature adulthood does not impair spatial learning in this task.

##### Probe Test

Adjusting for age and background, we detected no significant group differences in either of our measures of spatial recall during the memory probe test, when the platform was removed from the maze (Figures 3D,E; Sex × History ANCOVAs: for latency to former platform location, *p’s* > 0.23; for time spent in the NE quadrant that formerly contained the platform, *p’s* > 0.12). Thus, binge drinking during mature adulthood does not appear to impact the recall of spatial memory when assessed 24 h following training.

##### Reversal Test

Adjusting for age and background, we also detected no group differences in reversal learning in Experiment 1 (Figure 3F) [Trial effect: *F*_(3, 108)_ = 2.77, *p* = 0.045; other *p’s* > 0.27]. Thus, neither sex nor a history of binge drinking affects reversal learning in mature adult mice.

#### Radial Arm Maze

##### Acquisition Criterion

**Figure 4:**
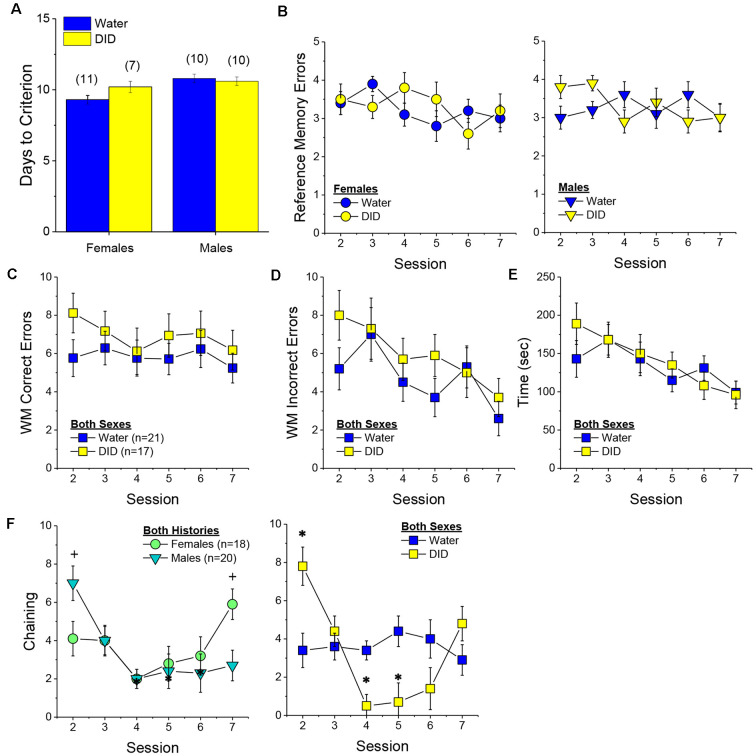
Effectsof binge drinking during later maturity (6–9 months of age) on radial arm maze performance in a heterogeneous cohort of mice. No effects of binge drinking (DID) were detected for the number of training days required to reach asymptotic performance in the radial arm maze **(A)**, the number of reference memory errors **(B)**, working memory (WM) correct errors **(C)**, WM memory incorrect errors **(D)** or the time taken **(E)** to navigate the radial arm maze over the first week of training. **(F)** Group differences were detected in the time-course of chaining behavior in Experiment 1; chaining decreased and increased linearly with training in males and females, respectively **(left)**, while chaining was stable in Water controls, it exhibited a U-shaped time-course in DID mice **(right)**. The data represent the means ± SEMs, adjusted for the covariates of age and background, of the number of mice indicated in parentheses. *p < 0.05 Water vs DID; ^+^*p* < 0.05 Male vs. Female (sex difference).

Adjusting for age and background, we detected a trend for a History × Sex interaction [*F*_(1, 37)_ = 3.05, *p* = 0.09] with respect to the number of sessions required for mature adult mice to reach asymptotic performance in the radial arm maze. As illustrated in Figure 4A, binge drinking females took slightly longer than their water controls to reach the acquisition criterion, with no difference observed in males. As some mice acquired the radial arm maze by the end of the 7th session, we examined for group differences only from the first week of this study (sessions 2–7).

##### Memory Errors and Time

When controlling for both age and background, we detected only a trend for a History × Session × Sex interaction for the number of reference memory errors committed across the first week of training in the radial arm maze [*F*_(5, 160)_ = 2.03, *p* = 0.08; other *p’s* > 0.20]. As illustrated, this interaction appeared to reflect relatively poorer reference memory performance by male binge drinking mice very early during training (Figure 4B; **right**), while female binge drinking mice tended to make more reference memory errors during the middle of the first week of training (Figure 4B; **right**). We detected no group differences in the number of working correct memory errors committed during the first 7 days of maze training (Figure 4C; History × Session × Sex ANCOVA, all *p’s* > 0.23). Although DID mice appeared to make more working memory incorrect errors in some trials than Water controls (Figure 4D), when adjusting for both age and background, this group difference was not statistically significant (History × Session × Sex ANCOVA, all *p’s* > 0.20). Likewise, we detected no group differences in the latency to complete the radial arm maze over the course of the first week of testing (Figure 4E; History × Session × Sex ANCOVA, all *p’s* > 0.21). Taken together, these results indicate that when the age and background of the mice are taken into account, binge drinking during mature adulthood does not impact reference or working memory in the radial arm maze.

##### Chaining

The failure to detect any binge drinking effect on our measures of radial arm maze performance prompted us to examine for differences in chaining behavior. Chaining refers to entries into adjacent arms and is a memory-independent navigation strategy that we have shown to be associated with anomalies in prefrontal cortex glutamate (Szumlinski et al., 2005). When controlling for both age and background, we detected a sex difference in the shape of the time-course of chaining behavior across the first week of training (Figure 4F, **left**) [*F*_(5, 160)_ = 2.88, *p* = 0.016], that reflected a relatively linear decline in chaining by males [linear test of within-subjects contrasts: *F*_(1, 16)_ = 3.94, *p* = 0.06], but a linear *increase* in chaining by females [linear test for within-subjects contrasts, *F*_(1, 16)_ = 6.15, *p* = 0.03]. As illustrated in Figure 4F, **(left)**, males exhibited more chaining than females on session 2 [*F*_(1, 37)_ = 5.85, *p* = 0.02], while females exhibited more chaining than males on session 7 [*F*_(1.37)_ = 6.86, *p* = 0.01]. These data suggest that male mice attempt and then discard chaining as a strategy for maze navigation, while females come to employ this non-memory strategy as training progresses. Of relevance to our hypothesis concerning the effects of binge drinking on cognition, we also detected a significant History × Session interaction (Figure 4F, **right**) [*F*_(5, 160)_ = 5.67, *p* < 0.0001], that reflected stable chaining behavior in Water mice (tests for within-subjects contrasts, all *p’s* > 0.64), but a U-shaped function in DID animals [quadratic test for within-subjects contrasts, *F*_(1, 14)_ = 6.43, *p* = 0.02], with DID mice exhibiting more chaining during session 2 [*F*_(1, 37)_ = 10.44, *p* = 0.003], but less chaining during session 4 [*F*_(1, 37)_ = 15.73, *p* < 0.0001] and session 5 [*F*_(1, 37)_ = 8.46, *p* = 0.006; other sessions, *p’s* ≥ 0.07], than Water controls. The U-shaped time-course of chaining behavior exhibited by DID mice suggests that they initially employ a non-memory strategy to navigate the maze, then discard this strategy relatively quickly (the next day) only to resume using this non-memory strategy later in training. Thus, mice with a history of binge drinking during mature adulthood are capable of adjusting their navigation strategy during radial arm maze training, indicative of intact cognitive flexibility, but a higher reliance on non-memory strategies for maze navigation.

### Experiment 2. Isogenic 6-Month-Old C57BL/6J (B6J) Mice

Statistical analyses of the data by ANCOVA, adjusting for the difference in time housed under a reverse light-cycle prior to the commencement of binge drinking procedures (1.5 months vs. 6 months), did not reveal any significant effect of this covariate for any of our measures (*p’s* > 0.15). Thus, all of the data from Experiment 2 are presented as unadjusted means ± SEMs and the statistical results reflect analyses of the unadjusted means.

#### Alcohol Intake and BECs

**Figure 5:**
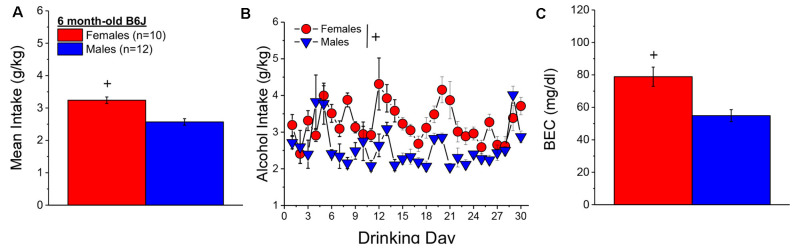
Bingedrinking during later maturity (6 months of age) in a cohort of isogenic C57BL/6J mice. **(A)** On average, females in Experiment 2 consumed more alcohol than males over the 30-day course of binge drinking. **(B)** Comparison of the time-courses of binge drinking between Experiment 2 male and female mice. **(C)** Consistent with the sex difference in binge drinking, BECs were higher in females vs. males in Experiment 2. The data represent the means ± SEMs of the number of mice indicated in parentheses in Panel **(A)**. ^+^*p* < 0.05 Females vs. Males.

Consistent with results from Experiment 1 (Figure 2), 6-month-old (a.k.a. mature adult) B6J females consumed significantly more alcohol than males over the 30-day drinking period (Figure 5A) [Sex effect: *F*_(1, 19)_ = 20.00, *p* < 0.0001] and a sex difference was detected in the time-course of alcohol intake (Figure 5B) [Sex × Day interaction: *F*_(29, 551)_ = 2.22, *p* < 0.0001]. Unexpectedly, the average alcohol intake of mature adult B6J mice of both sexes in Experiment 2 was approximately 1.5–2 g/kg lower than that reported previously by our group for 2 month-old adult B6J mice (Cozzoli et al., 2012; Lee et al., 2016; Szumlinski et al., 2019; Jimenez Chavez et al., 2020) and on the day of blood sampling, the alcohol intake of both the male and female B6J mice was, respectively, 2.34 ± 2.1 g/kg and 3.53 ± 0.41 g/kg [*t*_(20)_ = 2.90, *p* = 0.009]. Nevertheless, females exhibited a higher BEC than males [*t*_(20)_ = 3.45, *p* = 0.003] that was very close to the 80 mg/dl criterion for binge drinking (Figure 5C). In contrast, the BEC of the B6J males was well below the 80 mg/dl criterion, which is consistent with their relatively low alcohol intake on this day.

#### Morris Maze

##### Flag Test

**Figure 6:**
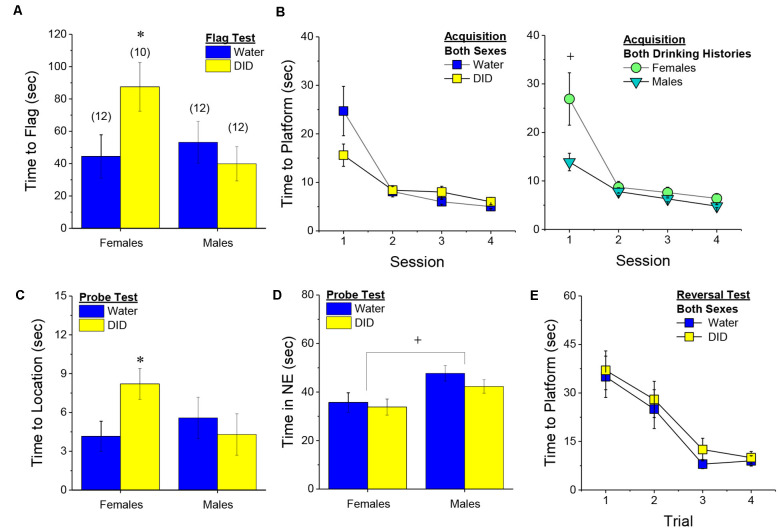
Effects of binge drinking during later maturity (6 months of age) on Morris water maze performance in cohorts of isogenic C57BL/6J mice. **(A)** Relative to water-drinking controls (Water), binge drinking (DID) females in Experiment 2 exhibited a longer latency to locate a visible, flagged, platform. **(B)** All mice readily acquired the location of a hidden platform in the Morris Maze over the 4-day course of training, although DID mice tended to locate the platform more quickly than Water controls on Session 1 **(left)**, and males were significantly quicker than females on Session 1 **(right)**. **(C)** On the probe test, female DID mice exhibited a longer latency to first enter the former location of the hidden platform. **(D)** No binge drinking effects were detected for the amount of time spent in the NE quadrant that formerly contained the hidden platform, although males spent more time, overall, than females. **(E)** No group differences were detected for reversal learning when the hidden platform was relocated within the maze. The data represent the means ± SEMs of the number of mice indicated in parentheses in Panel **(A)**. *p < 0.05 DID vs. Water; ^+^*p* < 0.05, Females vs. Males.

As observed in Experiment 1 (Figure 3A), a history of binge drinking impaired visually-cued localization of the platform in Experiment 2; however, this effect was detected only in female subjects (Figure 6A) [History × Sex interaction, *F*_(1, 45)_ = 0.70, *p* = 0.04; for female DID vs. Water, *t*_(20)_ = 2.15, *p* = 0.04; for male DID vs. Water, *t*_(22)_ = 0.79, *p* = 0.44]. There was a statistical trend towards a significant History × Sex interaction for the distance traveled by the mice during the flag test that paralleled the latency data (History × Sex: *p* = 0.08; data not shown), arguing further that the poor performance of the DID females on the flag test does not reflect an inability to swim or more immobility/floating. However, distinct from Experiment 1, we detected no difference in the number of Water vs. DID mice that failed to locate the flagged platform when both sexes were considered (5/24 water vs. 7/22 DID; *λ*^2^ = 0.57, *p* = 0.45). With the exception of 1 Water and 1 DID mouse, all of the B6J mice that required additional testing were able to locate the platform on the second trial, while the two remaining mice required a third trial before completing the task. While twice as many DID females failed to locate the platform on the first trial than female Water controls (6/10 DID vs. 3/12 Water), the proportion of female mice failing the task on the first attempt was also not statistically different (*λ*^2^ = 2.76, *p* = 0.10). No group difference was detected for the proportion of Water vs. DID males failing the task on the first trial (1/12 DID vs. 2/12 Water). These findings from Experiment 2 provide our first evidence that mature adult females may be more sensitive to the cognitive-impairing effects of alcohol than males.

##### Maze Training

Replicating Experiment 1, we detected a significant effect of binge drinking on the acquisition of the hidden platform location in the Morris water maze (Figure 6B) [History × Session: *F*_(3, 126)_ = 3.63, *p* = 0.02], that was sex-independent (History × Sex × Day, *p* = 0.13). As in Experiment 1 (Figure 3C), the History × Session interaction appeared to reflect poorer performance in the Water controls (Figure 6B, **left**), with *post hoc* comparisons failing to indicate specific Water-DID differences on any of the four training sessions (*p’s* > 0.09). Also akin to Experiment 1 (Figure 3D), we detected a significant Sex × Session interaction during maze training (Figure 6B, **right**) [*F*_(3, 126)_ = 3.25, *p* = 0.02]. As depicted in Figure 6B, **(right)**, the Sex × Session interaction reflected a longer latency of female mice to locate the hidden platform during Session 1 (tests for simple effects, for Session 1: *p* < 0.05; for Sessions 2–4; *p’s* > 0.05). These findings, derived from a homogenous cohort of 6-month-old mice, do not support any overt effect of binge drinking during mature adulthood on spatial learning in the Morris water maze.

##### Probe Test

In contrast to the results of Experiment 1, a significant History × Sex interaction was detected for the latency to first enter the platform’s former location in the study of isogenic B6J mice [*F*_(1, 45)_ = 4.25, *p* = 0.04]. This interaction reflected a significantly longer latency of DID females to enter the former platform location, relative to their Water controls (Figure 6C) [females: *t*_(20)_ = 2.16, *p* = 0.046; males: *p* = 0.45]. Females also spent less time than males in the NE quadrant that formerly contained the platform [Sex effect: *F*_(1, 45)_ = 9.16, *p* = 0.004], but this sex difference did not vary as a function of prior binge drinking history (Figure 6D; History effect and interactions, *p’s* > 0.28). These data from the probe test indicate that 6-month-old B6J females exhibit signs of poorer spatial recall than age-matched males, and appear to be more sensitive than males to certain memory-impairing effects of binge alcohol.

##### Reversal Test

As observed in Experiment 1, no group differences in reversal learning were detected in the 6-month-old B6J mice of Experiment 2 (Figure 6E) [Trial effect: *F*_(3, 126)_ = 20.34, *p* < 0.0001; all other *p’s* > 0.26]. Thus, binge drinking during mature adulthood does not impair reversal learning in the Morris water maze.

#### Radial Arm Maze

##### Acquisition

**Figure 7:**
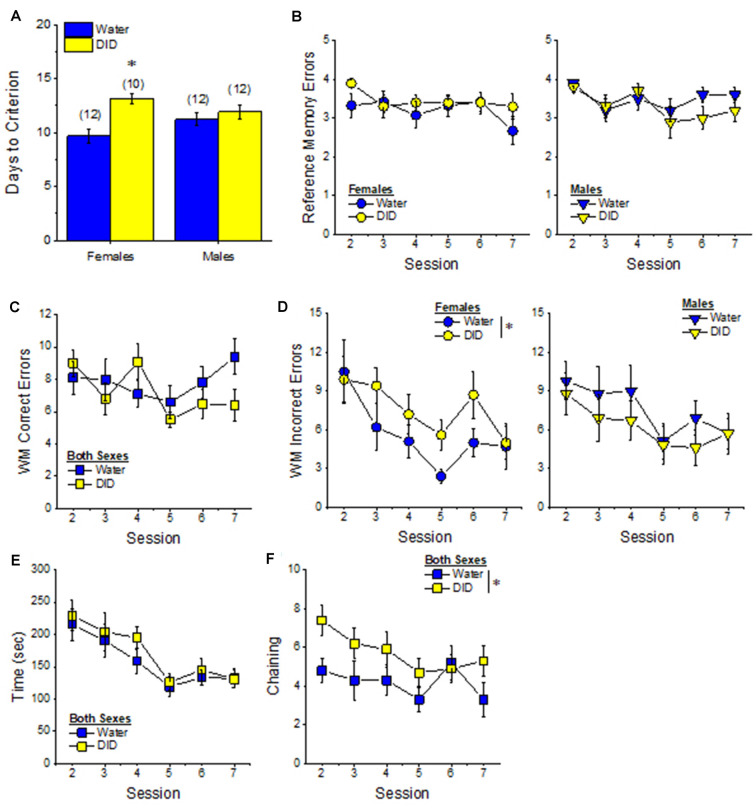
Effects of binge drinking during later maturity (6 months of age) on radial arm maze performance in cohorts of isogenic C57BL/6J mice. **(A)** The binge drinking (DID) female C57BL/6J mice in Experiment 2 required more time than their water-drinking (Water) controls to reach asymptotic performance in the radial arm maze. No significant effect of binge drinking was detected for the number of reference memory errors **(B)**, or working memory (WM) correct errors **(C)** committed over the first week of radial arm maze training. Female DID mice committed more WM incorrect errors than their Water controls **(D)**, but we detected no group differences in the time taken to navigate the maze **(E)**. **(F)** Finally, the DID mice in Experiment 2 exhibited more chaining, on average, than Water mice. The data represent the means ± SEMs of the number of mice indicated in parentheses in Panel **(A)**. *p < 0.05 DID vs. Water.

The pattern of group differences regarding the number of days required by 6-month-old B6J mice to reach asymptotic performance in the radial arm maze was very similar to that observed in Experiment 1 (Figure 4A vs. Figure 7A). However, in the case of the more homogeneous cohort of mice, the History × Sex interaction reached statistical significance [*F*_(1, 45)_ = 5.33, *p* = 0.03] and *post-hoc* tests confirmed that female DID mice required more days to acquire the maze than their Water controls [*t*_(20)_ = 4.30, *p* < 0.0001], with no DID-Water difference detected in males (Figure 7A; *p* = 0.38). These findings indicate that a history of binge drinking during mature adulthood impairs working memory performance selectively in female subjects.

##### Reference Memory Errors

In contrast to Experiment 1, no significant group differences were detected regarding the number of reference memory errors committed across the first week of training (Figure 7B), although a statistical trend was observed for a History × Sex interaction [Session effect: *F*_(5, 210)_ = 2.20, *p* = 0.05; History × Sex: *F*_(1, 42)_ = 3.26, *p* = 0.08; other *p’s* > 0.30]. An inspection of the data in Figure 7B suggested that this weak interaction reflected a tendency for DID females to make more reference memory errors during the first week of acquisition than their Water controls, while DID males tended to make fewer errors than their Water controls.

##### Working Memory Errors and Time

As reported for Experiment 1, we did not observe group differences in the number of working memory correct errors (Figure 7C) [Session effect: *F*_(5, 210)_ = 2.00, *p* = 0.08; other *p’s* > 0.28], but we detected a significant History × Sex interaction for the mean number of working memory incorrect errors committed over the course of the first 7 days of radial arm maze training [Session: *F*_(5, 210)_ = 5.74, *p* < 0.0001; History × Sex: *F*_(1, 41)_ = 4.21, *p* = 0.04]. As illustrated in Figure 7D, this interaction reflected a greater average number of working memory incorrect errors committed by female DID vs. Water mice [mean number of errors: for females, *t*_(20)_ = 2.73, *p* = 0.01; for males, *p* = 0.31]. Although DID females made more working memory incorrect errors than their Water counterparts, we detected no group differences in the latency of the 6-month-old mice to locate all four hidden platforms over the first week of training (Figure 7E) [Session: *F*_(5, 210)_ = 9.70, *p* < 0.0001; other *p’s* > 0.28]. These data further indicate that binge drinking during mature adulthood does not produce a global disruption of radial arm maze performance but rather induces specific impairments in working memory to which females appear to be more sensitive.

##### Chaining

Based on the chaining data from Experiment 1 (Figure 4F, **left**), we also examined for group differences in chaining behavior in Experiment 2. As illustrated in Figure 7F, we detected a significant overall History effect [*F*_(1, 41)_ = 4.19, *p* = 0.04], that reflected more chaining behavior in the DID vs. Water mice. However, in contrast to Experiment 1 (Figure 4F, **right**), we did not detect any sex differences in chaining behavior in Experiment 2 (History × Sex × Session ANOVA, other *p’s* > 0.18). These data provide additional evidence that mice with a history of binge drinking during mature adulthood employ a non-memory strategy to successfully navigate a radial arm maze.

### Experiment 3. Isogenic 6 and 18 Month-Old c57bl/6J (B6J) Mice

#### Alcohol Intake and BECs

**Figure 8:**
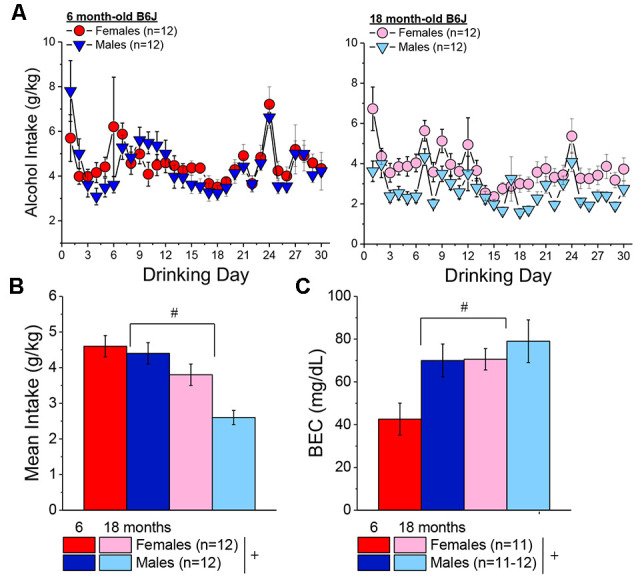
Bingedrinking during later maturity (6 months of age) vs. old-age (18 months of age) in isogenic C57BL/6J mice. **(A)** Comparison of the time-courses of binge drinking between the 6-month-old **(left)** and 18-month-old **(right)** mice in Experiment 3. **(B)** On average, females in Experiment 3 consumed more alcohol than males, and 6-month-old mice consumed more alcohol than 18-month-old mice, over the 30-day course of binge drinking. **(C)** With the exception of the female 6-month-old mice, the mice in Experiment 3 exhibited BECs within error of the 80 mg/dl criterion for binge drinking. The data represent the means ± SEMs of the number of mice indicated in parentheses in Panel **(A)**. ^+^*p* < 0.05 females vs. males; ^#^*p* < 0.05, 6 vs. 18 months old.

The time courses of alcohol intake by female and male 6 and 18-month-old mice in Experiment 3 are presented in Figure 8A. As expected based on the two prior experiments, females consumed more alcohol, on average, than males [Sex effect: *F*_(1, 44)_ = 5 0.71, *p* = 0.02]. Although this sex difference appeared to be more pronounced in the 18-month-old mice (Figure 8B), the interaction was only at trend level [Sex × Age, *p* = 0.09; Sex × Day, *p* = 0.56; Sex × Age × Day: *p* = 0.07]. Regardless of sex, the 6-month-old mice consumed more alcohol, on average, than the 18-month-old animals over the 30-day course of drinking (Figure 8B) [Age effect: *F*_(1, 44)_ = 21.12, *p* < 0.0001; Age × Day, *p* = 0.21].On the day of blood sampling (Day 30 in Figure 8A), we detected only an age-difference in alcohol intake [Age effect: *F*_(1, 47)_ = 23.72, *p* < 0.0001; Sex effect and interaction, *p’s* > 0.19]. Three statistical outliers were detected during the analyses of BECs that all exhibited very low BECs (i.e., <8 mg/dl) and included one 6 month female, one 18 month male and one 18 month female and their data were excluded from the statistical analysis of the BEC data. The BECs attained indicated both an age- and sex-difference (Figure 8C) [Sex effect: *F*_(1, 44)_ = 5.54, *p* = 0.02; Age effect: *F*_(1, 44)_ = 5.80, *p* = 0.02; interaction, *p* = 0.22] that reflected lower BECs in females vs. males and in the 6-month-old vs. 18-month-old mice. As depicted in Figure 8C, the BECs of the 18-month-old mice and the 6-month-old males were within error of the 80 mg/dl criterion for binge drinking, while that of the 6-month-old females was inexplicably half that exhibited by the other groups.

#### Behavioral Test Battery of Negative Effect

Younger adult (i.e., 2 months-old) male and female mice exhibit negative effect when assayed 24 h following a period of binge drinking that includes panic-like swimming behavior in the forced swim test (e.g., Lee et al., 2015; Szumlinski et al., 2019; Jimenez Chavez et al., 2020). To address the possibility that the relatively poor ability of DID mice to locate a visually-cued flagged platform in Experiments 1 and 2 might reflect, in some part, withdrawal-induced anxiety, we determined the effective phenotype of the Water and DID mice from Experiment 3, 1 day following their last 30-day drinking session using a behavioral test battery for negative effect similar to that employed in our prior work.

##### Acoustic Startle and PPI

**Figure 9:**
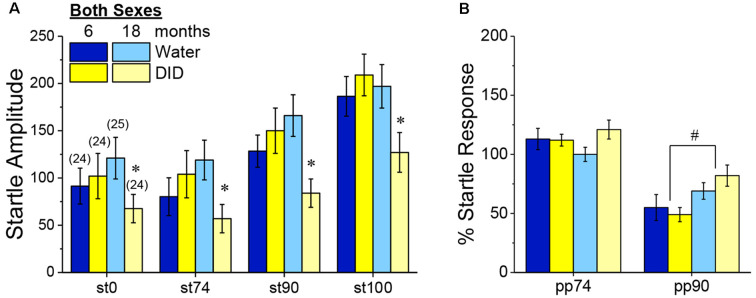
Effects of binge drinking during later maturity (6 months of age) or old age (18 months of age) on acoustic startle in isogenic C57BL/6J mice. **(A)** Relative to water-drinking controls (Water), binge drinking (DID) 18-month-old mice exhibited lower acoustic startle, with no binge drinking effect apparent in 6-month-old mice. **(B)** Only the 90 dB prepulse inhibited acoustic startle and the extent of this inhibition was larger in 6 vs. 18-month-old mice, with no effect of binge drinking detected for the acoustic startle. The data represent the means ± SEMs of the number of mice indicated in parentheses in Panel **(A)**. *p < 0.05 DID vs. Water; ^#^*p* < 0.05 6 vs. 18 months old.

Although B6J mice are well-characterized to exhibit age-related hearing loss (e.g., Henry and Chole, 1980), all B6J mice in Experiment 3 exhibited a tone-dependent increase in their magnitude of acoustic startle that was age-independent (Figure 9A) [Tone effect: *F*_(3, 270)_ = 57.70, p 0.0001; no Age effect, no Sex effect and no Age or Sex × Tone interactions, *p’s* > 0.50]. Curiously, we did detect an overall Age × History interaction [*F*_(1, 90)_ = 5.30, *p* = 0.02], that reflected significantly lower startle, overall, in 18-month-old DID mice, relative to their Water controls (Figure 9A) [*t*_(48)_ = 2.68, *p* = 0.01]. However, consistent with our prior studies of 2 month-old adult mice (e.g., Lee et al., 2015), we detected no effect of binge drinking on acoustic startle in the 6-month-old mice in Experiment 3 (Figure 9A; *t*-test, *p* = 0.47).

Consistent with our published work in 2 month-old adult B6J mice (e.g., Lee et al., 2015), only the 90 dB pre-pulse effectively reduced the magnitude of acoustic startle in Experiment 3 (Figure 9B) [PPI effect: *F*_(1, 90)_ = 132.12, *p* < 0.0001]. Perhaps not surprising given their advanced age, 18-month-old mice exhibited weaker PPI at the 90 dB pre-pulse than their 6-month-old counterparts (Figure 9B) [Age × Prepulse: *F*_(1, 270)_ = 9.55, *p* = 0.003; for 90 dB, *t*_(96)_ = 2.77, *p* = 0.007; for 75 dB, *p* = 0.74]. Although binge drinking history reduced startle amplitude in the aged mice (Figure 9A), it did not alter the capacity of the 90 dB pre-pulse to inhibit acoustic startle in either sex (Figure 9B; no History effect or interactions, *p’s* > 0.14; no sex effect or interactions, *p’s* > 0.06). These PPI data indicate that a recent binge drinking history does not impair sensorimotor gating in mature adult mice nor does it exacerbate the age-related decline in sensorimotor gating.

##### Light-Dark Shuttle Box

**Figure 10:**
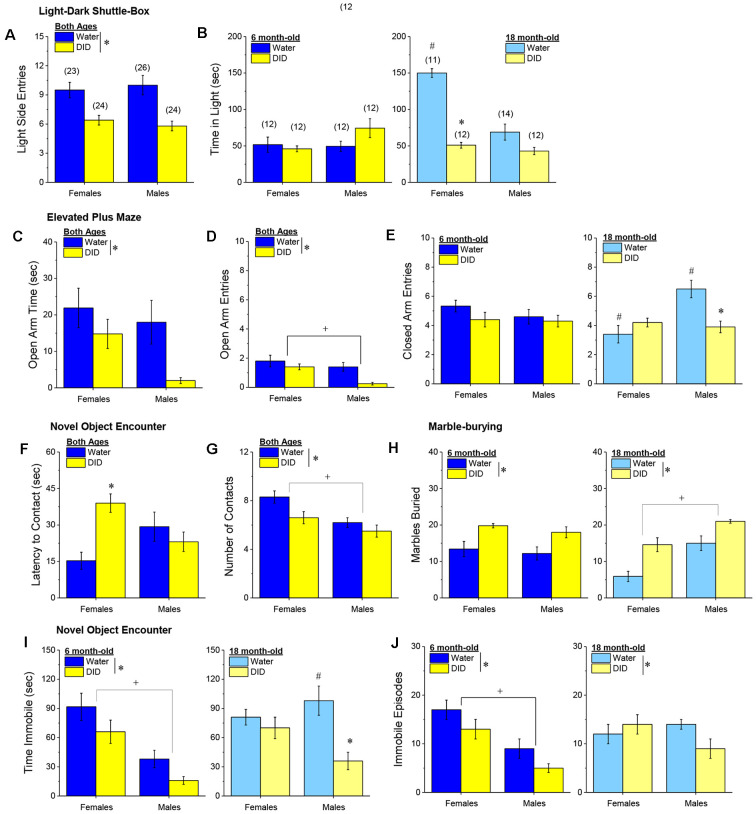
Effectsof binge drinking during later maturity (6 months of age) or old age(18 months of age) on behavioral indices of negative affect inisogenic C57BL/6J mice. **(A)** Regardless of age or sex, binge drinking (DID) lowered the time spent in the light-side of a light-dark shuttle-box, relative to water-drinking controls (Water). **(B)** Only 18-month-old DID females spent less time than their Water controls in the light-side of the light-dark box. In the elevated plus-maze, DID mice spent less time in **(C)** and made fewer entries into **(D)**, the open arm, with males making fewer open arm entries than females, irrespective of drinking history or age. **(E)** Binge drinking reduced the number of closed arm entries only in male 18-month-old mice. In the novel object encounter test, female DID mice exhibited a longer latency to approach the novel object **(F)**, and although males approached the novel object less often that females, both male and female DID mice made fewer contacts with the novel object than Water controls **(G)**. **(H)** Irrespective of age, DID mice buried more marbles than Water controls, with 18-month-old males burying more marbles than their female counterparts. **(I)** Both 6-month-old DID mice **(left)** and male 18-month-old mice **(right)** spent less time immobile in the forced swim test. **(J)** A similar pattern of effect was observed with respect to the number of immobile episodes, although the main effect of binge drinking was detected for this variable. The data represent the means ± SEMs of the number of mice indicated in parentheses in Panel **(B)**. *p < 0.05, DID vs. Water; ^+^*p* < 0.05, Females vs. Males; ^#^*p* < 0.05, 6 vs. 18-month-olds.

We did not detect any age-related differences with respect to the number of entries into the light-side of the light-dark shuttle box (age effect and interactions, *p’s* > 0.25), with both male and female DID mice making fewer light-side entries, relative to their water-drinking controls (Figure 10A) [History effect: *F*_(1, 96)_ = 24.45, *p* < 0.0001; Sex effect and interactions, *p’s* > 0.25). However, a significant three-way interaction was detected for the time spent in the light side [*F*_(1, 96)_ = 14.41, *p* < 0.0001]. Deconstruction of the interaction along the age factor revealed no group differences in the 6-month-old mice (Figure 10B, **left**; Sex × History ANOVA, *p’s* > 0.10), while a Sex × History interaction was detected in the 18-month-old mice [*F*_(1, 48)_ = 21.73, *p* < 0.0001]. As illustrated in Figure 10B, **right**, this interaction reflected, in part, a marked reduction in time spent in the light-side by 18-month-old DID females, relative to their Water controls [*t*_(21)_ = 13.00, *p* < 0.0001], with a non-significant Water-DID difference observed in 18-month-old males (*t*-test, *p* = 0.06). Deconstruction of the three-way interaction along the history factor also revealed a significant Age × Sex interaction in Water controls [*F*_(1, 48)_ = 22.76, *p* < 0.0001] that was not apparent in DID mice (ANOVA, all *p’s* > 0.27). As illustrated in Figure 10B **(left vs. right)**, 18-month-old Water female mice spent considerably more time in the light-side than their 6-month-old counterparts [*t*_(21)_ = 7.88, *p* < 0.0001], while no age-related difference was observed in Water control males (*t*-test, *p* = 0.76). Taken together, these data extend to older mice the results of prior work in younger adult (2-month-old) mice (e.g., Lee et al., 2015) indicating that early withdrawal from a month-long history of binge drinking produces an anxiogenic phenotype in the light-dark shuttle box. Moreover, these data argue that a history of binge drinking occludes what appears to be an age-related decline in baseline anxiety-like behavior in female mice, increasing the magnitude of the withdrawal-induced anxiogenic state to a greater extent in aged females than in aged males.

##### Elevated Plus Maze

Age did not impact the time spent in the open arm of the elevated plus maze (Figure 10C; no age effect or interactions, *p’s* > 0.34) nor the number of open arm entries (Figure 10D; no Age effect or interactions, *p’s* > 0.21). However, for both indices of anxiety-like behavior, we detected a main history effect [for time in open arm: *F*_(1, 96)_ = 6.14, *p* = 0.014; for open arm entries: *F*_(1, 96)_ = 8.31, *p* = 0.005], which reflected higher anxiety-like behavior in DID vs. Water mice. Although DID males exhibited the lowest level of open arm exploration of the groups tested (Figures 10C,D), data analyses indicated a main sex effect only for the number of open arm entries [*F*_(1, 96)_ = 7.94, *p* = 0.006; for time in open arm: *F*_(1, 96)_ = 3.53, *p* = 0.06, n.s.], with no interactions detected with the sex factor for either variable (all *p’s* > 0.20). Thus, in the elevated plus maze, a binge drinking history produces a negative effect state that is comparable both between male and female subjects, as well as between mature adult and old mice.

The number of closed arm entries was also examined as a gross index of general motor activity in the elevated plus maze and analysis revealed a significant Sex × History × Age interaction [*F*_(1, 96)_ = 9.99, *p* = 0.002]. Deconstruction of this interaction along the Age factor revealed no group differences for the 6-month-old mice (Figure 10E, **left**; Sex × History ANOVA, *p’s* > 0.21), but a male-selective reduction in close arm entries in 18-month-old DID mice (Figure 10E, **right**) [Sex × History: *F*_(1, 48)_ = 13.21, *p* = 0.001; for 18-month males, *t*_(24)_ = 3.50, *p* = 0.002; for 18-month females, *p* = 0.12]. Deconstruction of the three-way interaction along the history factor revealed a significant Age × Sex interaction in Water controls [*F*_(1, 48)_ = 12.66, *p* = 0.001] that was not apparent in DID mice (all *p’s* > 0.47). In the case of closed arm entries, 18-month-old female and male water controls exhibited, respectively, less and more closed arm activity than their 6-month-old counterparts (Figure 10E, **left vs. right**) [for females, *t*_(21)_ = 2.81, *p* = 0.01; for males, *t*_(24)_ = 2.34, *p* = 0.03]. These latter data indicate that the effects of binge drinking on anxiety-like behavior in the elevated plus maze can be dissociated from effects on general activity, with respect to both sex and the age of the mice.

##### Novel Object Encounter Test

Irrespective of age, female DID mice exhibited the longest latency to first approach a novel object (Figure 10F) [Sex × History: *F*_(1, 96)_ = 10.26, *p* = 0.002; History effect: *p* = 0.06; other *p’s* > 0.18], with a significant Water-DID difference confirmed in female [*t*_(45)_ = 4.49 *p* < 0.0001], but not in male, mice (*t*-test, *p* = 0.40). Also irrespective of age, a history of binge drinking reduced the number of novel object contacts [History effect: *F*_(1, 96)_ = 5.14, *p* = 0.03; no Age effects or interactions, *p’s* > 0.28]. Although an inspection of Figure 10G suggested that the alcohol drinking-induced reduction was larger in females than in males, this was not supported by the statistical analyses of the data [Sex effect: *F*_(1, 96)_ = 9.20, *p* = 0.003; other *p’s* > 0.19]. Thus, in the novel object encounter test, both 6 and 18-month-old females exhibit more signs of withdrawal-induced anxiety-like behavior than males.

##### Marble-Burying Test

A history of binge drinking significantly increased the number of marbles buried, irrespective of either sex or age (Figure 10H) [History effect: *F*_(1, 96)_ = 36.08, *p* < 0.0001; no interactions with the history factor, *p’s* > 0.43]. This overall binge drinking effect aligns with the results of the elevated plus maze, in which withdrawal-induced anxiety was both sex- and age-independent (Figures 10C,D). However, aligning with the data for the light-dark box (Figure 10B), we detected a robust Age × Sex interaction for marble-burying [Age × Sex: *F*_(1, 96)_ = 18, 02, *p* < 0.0001]. As illustrated in Figure 10H, **right**, the Age × Sex interaction reflected higher anxiety-like behavior in 18 month-old males vs. females [*t*_(47)_ = 8.77, *p* < 0.0001], with no sex difference in marble-burying apparent in 6-month-old mice (Figure 10H, **left**; *t*-test, *p* = 0.49). These marble-burying data provide additional evidence that females exhibit an age-dependent reduction in anxiety-like behavior, but that binge drinking history induces an anxiety-like state, irrespective of the age or sex of mice.

##### Forced Swim Test

Marked group differences were detected with respect to the time spent immobile in the forced swim test [Sex × Age × History: *F*_(1, 96)_ = 4.72, *p* = 0.04]. Deconstruction of the three-way interaction along the age factor revealed that 6-month-old females spent more time immobile than males (Figure 10I, **left**) [Sex effect: *F*_(1, 47)_ = 25.25, *p* < 0.0001; interaction, *p* = 0.88], and DID mice of both sexes spent less time immobile than Water controls [History effect: *F*_(1, 47)_ = 5.34, *p* = 0.03]. Comparable analyses of the data from the 18 month-old mice indicated a significant Sex × History interaction [*F*_(1, 47)_ = 4.81, *p* = 0.03], that reflected less immobility in DID males vs. their water controls (Figure 10I, **right**) [for males, *t*_(24)_ = 3.54, *p* = 0.002; for females, *p* = 0.41]. Deconstruction of the three-way interaction along the History factor indicated a significant Sex × Age interaction for Water controls [*F*_(1, 47)_ = 7.95, *p* = 0.007] that reflected an age-dependent increase in the time spent immobile by male, but not by female, subjects [for males, *t*_(24)_ = 3.36, *p* = 0.003; for females, *p* = 0.56]. In contrast, no age-dependent change in immobility was detected in DID mice (Age effect and interaction, *p’s* > 0.20), with females spending more time immobile overall than males [Sex effect: *F*_(1, 47)_ = 20.65, *p* < 0.0001].

A similar pattern of results was found with respect to the number of immobile episodes in the forced swim test. Overall, binge drinking history reduced the number of immobile episodes, irrespective of the age or sex of the mice [History effect: *F*_(1, 95)_ = 5.76, *p* = 0.019]. Although inspection of Figure 10J suggested that 18-month-old females were insensitive to the binge drinking-induced reduction in immobility, we detected no significant interactions with the history factor (*p’s* > 0.10). That being said, we did detect a significant Sex × Age interaction [*F*_(1, 95)_ = 6.94, *p* = 0.01] that reflected more immobility in 6-month-old females than males (Figure 10J, **left**) [*t*_(46)_ = 4.50, *p* < 0.0001], with no sex difference apparent in 18-month-old mice (Figure 10J, **right**; *t*-test, *p* = 0.27). Thus, as reported for younger adult (2-month-old) mice (e.g., Lee et al., 2015), a history of binge drinking increases swimming behavior in both mature adult and old mice in the forced swim test. Given that pretreatment with anxiolytic drugs attenuates this effect of alcohol withdrawal (at least in younger adult mice; Lee et al., 2017a), we consider these forced swim data in line with the results from the other more traditional assays of anxiety-like behavior as indicating that binge drinking during later life induces an anxiety-like state, the magnitude of which can vary with sex and age of drinking onset.

#### Morris Water Maze

**Figure 11:**
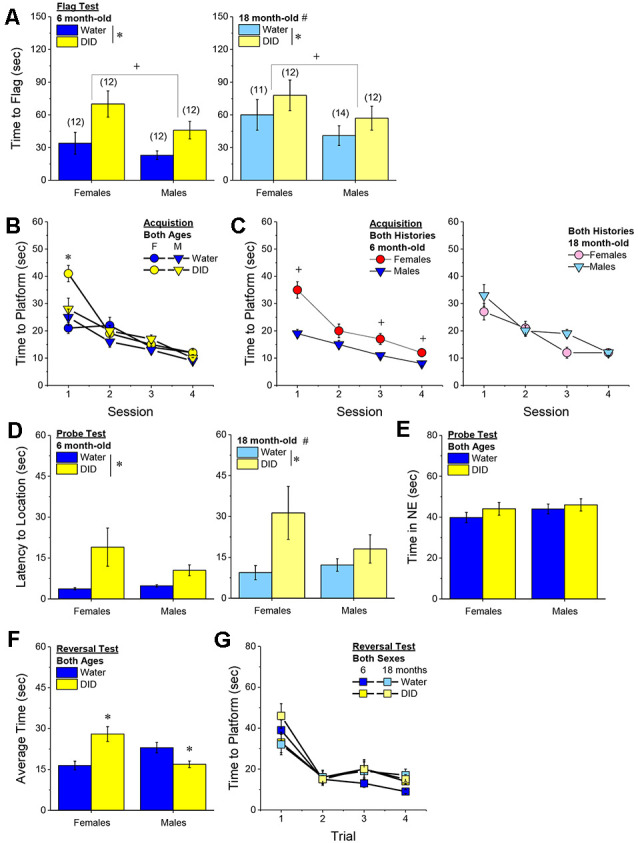
Effectsof binge drinking during later maturity (6 months of age) or old age (18 months of age) on Morris water maze performance in isogenic C57BL/6J mice. **(A)** Relative to water-drinking controls (Water), binge drinking (DID)mice in Experiment 3 exhibited a longer latency to locate a visible, flagged, platform, with 18-month-old mice and females requiring more time than 6 month-mice and males to locate the platform. **(B)** All mice readily acquired the location of a hidden platform in the Morris Maze over the 4-day course of training, although female DID mice required more time to locate the platform on Session 1. **(C)** Regardless of drinking history, female 6-month-old mice required more time than males to locate the hidden platform during maze acquisition **(left)**, with no sex difference detected in 18-month-old mice **(right)**. **(D)** On the probe test, DID mice exhibited a longer latency to first enter the former location of the hidden platform, irrespective of age or sex, although 18-month-old mice were more impaired, overall, than 6-month-old mice. **(E)** No binge drinking effects were detected for the amount of time spent in the NE quadrant that formerly contained the hidden platform, although males spent more time, overall, than females. **(F)** On the reversal test, female DID mice of both ages required more time, on average, to locate the relocated platform than their Water controls, while male DID mice spent less time than Water controls. **(G)** No overt group differences were detected for the time-course of reversal learning when the hidden platform was relocated within the maze. The data represent the means ± SEMs of the number of mice indicated in parentheses in Panel **(A)**. *p < 0.05, DID vs. Water; ^+^*p* < 0.05, Females vs. Males; ^#^*p* < 0.05, 6 vs. 18-month-olds.

Not surprisingly, 18-month-old mice took longer to locate the flagged platform than did the 6-month-old mice [Age effect: *F*_(1, 96)_ = 4.18, *p* = 0.04; Sex effect: *F*_(1, 96)_ = 5.93, *p* = 0.02]. Although it appeared that a history of binge drinking increased the time to locate the flagged platform more so in 6-month-old animals (Figure 11A, **left**) than in 18-month-old animals (Figure 11A, **right**), the results of the statistical analyses did not support this observation [History effect: *F*_(1, 96)_ = 9.24, *p* = 0.018; all other *p’s* > 0.42]. Thus, the magnitude of the alcohol-induced impairment in visually-cued navigation did not vary significantly as a function of aging. An inspection of Figure 11A, suggested that the 6-month-old DID mice exhibited a level of behavioral impairment on the Flag test comparable to that exhibited by aged Water controls. To test this notion, planned comparisons were conducted between the DID 6-month-old mice and sex-matched, 18-month-old, Water controls. This analysis confirmed a comparable level of impairment in both males and females [for females, *t*_(21)_ = 0.58, *p* = 0.57; for males, *t*_(24)_ = 0.36, *p* = 0.73]. These findings replicate the results of the prior two studies, indicating that binge drinking impairs the performance of mature adult mice on a visually-cued spatial navigation task, extend these findings to old mice and provide evidence that binge drinking during mature adulthood advances the onset of this particular cognitive impairment in both male and female mice.

##### Training

Analyses of the time taken to locate the hidden platform in the Morris water mazeindicated that sex significantly influenced the effect of binge drinking on learning [Sex × History × Session: *F*_(3, 267)_ = 8.67, *p* < 0.0001; 4-way interaction, *p* = 0.50]. Deconstruction this is three-way interaction along the Sex factor revealed a significant Drinking × Session interaction for female mice [*F*_(3, 135)_ = 14.76, *p* < 0.0001], that reflected a longer latency for DID vs. Water females to locate the hidden platform on Session 1 of training (Figure 11B) [for session 1, *t*_(45)_ = 5.66, *p* < 0.0001; for other sessions, *t’s* < 0.80, *p’s* > 0.43]. In contrast, binge drinking history did not affect learning in males (Figure 11B) [Session effect: *F*_(3, 144)_ = 28.84, *p* < 0.0001; other *p’s* > 0.14]. The Age × History × Session interaction was shy of statistical significance (*p* = 0.09), indicating that the drinking-induced impairment in spatial learning was not significantly different between 6 and 18-month-old mice. However, we did detect a significant Age × Sex × Session interaction [*F*_(3, 267)_ = 5.35, *p* = 0.001] for spatial learning that reflected poorer spatial learning by female vs. male 6-month-old mice (Figure 11C, **left**) [Sex × Session: *F*_(3, 138)_ = 4.49, *p* = 0.005; for Sessions 1, 3, 4, *t*_(46)_ > 2.50, *p’s* < 0.02; for trial 2, *p* = 0.08], but no sex difference in the learning for 18-month-old animals (Figure 11C, **right**) [Session effect: *F*_(3, 141)_ = 27.04, *p* < 0.0001; no other *p’s* > 0.15].

#### Probe Test

Irrespective of sex, 18-month-old mice exhibited a longer latency to first enter the former platform location than younger mice (Figure 11D, **left vs. right**) [Age effect: *F*_(1, 96)_ = 6.08, *p* = 0.02; no Age × Sex interactions, *p’s* > 0.60], indicative of an age-related impairment in spatial recall. However, irrespective of age, DID mice exhibited a longer latency to first enter the former platform location [History: *F*_(1, 96)_ = 13.04, *p* = 0.001]. Although inspection of Figure 11D suggested that the binge drinking effect was more robust in females than in males, the Sex × Drinking interaction was not statistically significant (*p* = 0.06), owing likely to the larger variability in the data for females of both ages. To test the hypothesis that a binge drinking history elicited a comparable impairment in this measure of spatial recall as that produced by normal aging, we conducted planned comparisons between the 6-month-old DID mice and the 18-month-old Water controls. As suggested by Figure 11D **(left vs. right)**, this comparison revealed no statistically significant difference [*t*_(47)_ = 0.93, *p* = 0.36], indicating that binge drinking during mature adulthood accelerates impairments in the spatial recall. However, in line with Experiment 2, we detected no group differences for the total time spent in the former platform quadrant (Figure 11E; Age × Sex × Drinking ANOVA, all *p’s* > 0.26). Taken all together, the results from our two studies of B6J mice argue that the latency to the platform location is more sensitive to the recall-impairing effects of alcohol than the more classic dependent variable of time spent in the former platform quadrant.

#### Reversal Test

An analysis of the time taken to find the relocated platform during the reversal test indicated a significant overall Sex × History interaction [*F*_(1, 89)_ = 20.26, *p* < 0.0001], that reflected a longer and shorter average latency, respectively, for DID vs. Water female and male mice to find the relocated platform over the four training trials (Figure 11F) [for females, *t*_(45)_ = 3.63, *p* = 0.001; for males, *t*_(48)_ = 2.66, *p* = 0.01]. Thus, regardless of the age of drinking onset, the female mice in this study were more sensitive overall to an alcohol-induced impairment in reversal learning. This being said, we also detected a significant Age × History × Trial interaction [*F*_(13, 267)_ = 3.20, *p* = 0.02; four-way interaction, *p* = 0.11]. As illustrated in Figure 11G, this interaction appeared to reflect, in part, group differences in the time taken to locate the relocated platform on trial 1, as well as faster latencies by 6-month-DID mice to locate the relocated platform on trials 3 and 4, relative to the other groups. However, when the data were collapsed across sex, deconstruction of this interaction along the age, history or trial factors did not yield significant group differences (History × Trial ANOVAs for 6 vs. 18-month-old mice: *p’s* > 0.06; Age × Trial ANOVAs for Water vs. DID mice: *p’s* > 0.14; Age × History univariate ANOVAs conducted separately on trial 1, 2, 3 or 4: *p’s* > 0.06]. Taken together, the data in Figure 11F, coupled with the negative results of these *post hoc* analyses, indicate that the alcohol-induced impairment in reversal learning is age-independent.

#### Radial Arm Maze

##### Acquisition

**Figure 12:**
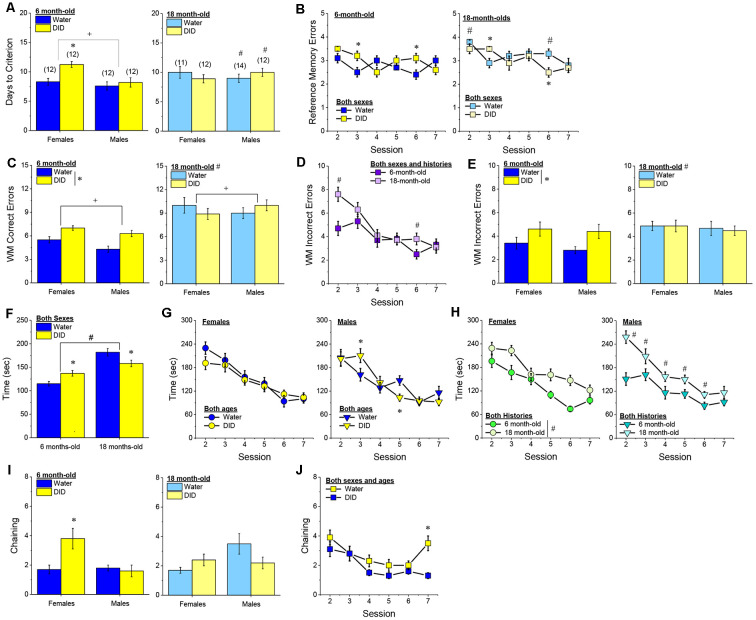
Effectsof binge drinking during later maturity (6 months of age) and old age (18 months of age) on radial arm maze performance in isogenic C57BL/6J mice. **(A)** The 6 month-old, binge drinking (DID), female C57BL/6J mice in Experiment 3 required more time than their water-drinking (Water) controls to reach asymptotic performance in the radial arm maze, with 18-month-old males also requiring more time than their 6-month-old counterparts, regardless of DID status. **(B)** A complex interaction between age and binge drinking history was observed for the change in reference memory errors during the first week of radial arm maze testing, that reflected a more consistent tendency for 6-month-old DID mice to commit more errors than their Water controls. **(C)** Although 18-month-old mice committed more working memory correct errors, on average, than the younger mice, only 6-month-old DID mice committed more of this error type than their water controls. **(D)** Likewise, 18-month-old mice committed more working memory incorrect errors than 6-month-old mice during radial arm maze training, however **(E)** only 6-month-old DID mice committed more working memory incorrect errors than their Water controls. **(F)** No significant effect of binge drinking was detected for the number of reference memory errors **(B)**, or working memory (WM) correct errors **(C)** committed over the first week of radial arm maze training. Female DID mice committed more WM incorrect errors than their Water controls **(D)**, but we detected no group differences in the time taken to navigate the maze **(E)**. **(F)** Although 18-month-old mice required more time, on average than 6-month-old mice to navigate the maze, 6 and 18-month-old DID mice required more and less time, respectively, to complete the maze than their Water controls. **(G)** Binge drinking altered the time-course of radial arm maze learning selectively in male mice and **(H)** a modest, but significant, sex difference was detected for the impaired radial arm maze learning exhibited by female **(left)** and male **(right)** 18-month-old mice. **(I)** Regardless of age, female DID mice exhibited more chaining behavior, on average than their exhibited impaired radial arm maze learning. **(J)** When the time-course of chaining is considered, both male and female mice exhibited more chaining behavior on the 7th training session than Water controls. The data represent the means ± SEMs of the number of mice indicated in parentheses in Panel **(A)**. *p < 0.05, DID vs. Water; ^+^*p* < 0.05, Female vs. Male; ^#^*p* < 0.05, 6 vs. 18-month-olds.

In Experiment 3, a prior history of binge drinking appeared to selectively increase the number of days required by 6-month-old female mice to reach asymptotic performance in the radial arm maze (Figure 12A). This observation was supported by a significant three-way interaction for this variable [*F*_(1, 95)_ = 5.00, *p* = 0.03], the deconstruction of the interaction along the Sex factor [for males, no History effect or interaction, *p’s* > 0.30; for females, Age × Drinking History: *F*_(1, 46)_ = 9.29, *p* = 0.004] and *post hoc* comparisons between Water and DID females at each age group [for 6-month-old females, *t*_(22)_ = 3.94, *p* = 0.0001; for 18-month-old females, *p* = 0.28]. In fact, the magnitude of the binge drinking effect in the younger, 6-month-old, female mice obscured the detection of an age-dependent increase in the time taken to acquire the radial arm maze in female subjects in this study [for females, Age effect: *p* = 0.83], which was readily apparent in male mice [age effect: *F*_(1, 48)_ = 4.76, *p* = 0.03]. These findings with respect to the number of days required to reach the acquisition criterion in the radial arm maze replicate the results of Experiment 2 and further the observation that a history of binge drinking during mature adulthood produces a female-selective impairment in working memory performance, with little evidence for an alcohol-induced perturbation in maze learning in more aged mice.

##### Reference Memory Errors

Irrespective of sex, 18-month-old mice made significantly more reference memory errors during the first week of radial arm maze training than their 6-month-old counterparts (Figure 12B; **left vs. right**) [Age effect: *F*_(1, 88)_ = 9.93, *p* = 0.002; Age × Session, *p* = 0.49; no Sex effect or interactions, *p’s* > 0.26] and binge drinking differentially modified the time-course of reference memory errors in 6- vs. 18-month-old mice [Age × History × Session: *F*_(5, 440)_ = 4.83, *p* < 0.0001]. Deconstruction of this three-way interaction along the session factor indicated that 18-month-old Water controls committed more reference memory errors on session 2 than their 6-month-old counterparts [*F*_(3, 95)_ = 3.91, *p* = 0.001; LSD *post-hoc* tests, 6- vs. 18-month water: *p* = 0.001], with no age-related difference in reference errors detected in DID mice (*p* = 0.85). On session 3, both 6 and 18-month-old DID mice committed more reference memory errors than their Water controls [*F*_(3, 95)_ = 7.09, *p* < 0.0001; LSD *post-hoc* tests, for 6-month Water vs. DID, *p* = 0.003; for 18-month Water vs. DID, *p* = 0.01]. No group differences were detected during sessions 4, 5 or 7 (one-way ANOVA, *p’s* > 0.10), while several group differences were noted on session 6 [*F*_(3, 95)_ = 4.75, *p* = 0.004], including a greater number of reference errors by 18 vs. 6-month-old Water controls (*p* = 0.03), and by 6-month-old DID mice vs. their Water controls (*p* = 0.02). Addition, as well as fewer reference memory errors by 18-month-old DID mice vs. their Water controls (*p* = 0.005). Taken together, these data for reference memory errors provide some evidence that binge drinking history can influence spatial reference memory also in the radial arm maze, with 6-month-old mice exhibiting more consistent signs of impairment than aged mice.

##### Working Memory Errors

Analysis of the number of working memory correct errors committed by the mice in Experiment 3 indicated more errors overall in females vs. male subjects [Sex effect: *F*_(1, 88)_ = 5.36, *p* = 0.02]. Despite this, we detected no significant interactions with the Sex factor on this measure (Age × Sex × History, *p* = 0.10; other *p’s* > 0.34). Inspection of Figure 12C suggested that 18-month-old mice committed more working memory correct errors than their 6-month-old counterparts; however, the Age effect was not statistically significant (*p* = 0.75). We did detect a significant Age × History interaction [Age × History: *F*_(1, 88)_ = 9.89, *p* = 0.002], that reflected more working memory correct errors in 6-month-old DID mice vs. their water controls [*t*_(46)_ = 4.56, *p* < 0.0001], with no binge drinking effect apparent in 18-month-old mice (*t*-test, *p* = 0.95). Planned comparisons conducted between the number of working memory correct errors committed by 6-month-old DID and 18-month-old Water controls confirmed that the alcohol-induced working memory impairment exhibited by the younger mice was comparable to that of aged animals (Figure 12C, **left vs. right**) [*t*_(46)_ = 1.91, *p* = 0.06].

An examination of the time-course of the number of working memory incorrect errors committed during the first week of radial arm maze training indicated that, irrespective of sex and drinking history, 18-month-old mice committed more errors than their 6-month-old counterparts, notably on sessions 2 and 6 of training (Figure 12D) [Age × Session: *F*_(5, 440)_ = 2.78, *p* = 0.02; tests for main effects, for session 2, *p* = 0.002; for session 6, *p* = 0.05; for session 3, *p* = 0.07; for other sessions, *p’s* > 53; no other session interactions, *p’s* > 0.1; no Sex effect or interactions, *p’s* > 0.40]. Also irrespective of sex, binge drinking history increased the average number of working memory errors committed by 6-month-old mice during the first week of training (Figure 12E, **left**), with no binge drinking effect detected in 18-month-old animals (Figure 12E, **right**) [Age × Drinking History: *F*_(1, 88)_ = 4.44, *p* = 0.04; for 6-month-old, *t*_(46)_ = 2.85, *p* = 0.007; for 18-month-old, *p* = 0.88]. As observed for working memory correct errors (Figure 12C), the average number of working memory incorrect errors committed by 6-month-old DID mice was comparable to that exhibited by the 18-month-old Water controls (Figure 12E, **left vs. right**) [*t*_(46)_ = 0.59, *p* = 0.56], providing further evidence that a history of binge drinking during mature adulthood accelerates the onset of working memory impairments.

##### Time

In contrast to the results of Experiment 2, an analysis of the amount of time taken for 6 and 18-month-old mice to complete the radial arm maze during the first week of training revealed an overall Age × Drinking History interaction [*F*_(1, 88)_ = 11.47, *p* = 0.001]. Although 18-month-old mice required more time to navigate the radial arm maze than the younger mice, overall (Figure 12F) [Age effect: *F*_(1, 88)_ = 44.56, *p* < 0.0001], 6-month-old DID mice took longer to navigate the maze, relative to their Water controls, while the opposite pattern of effect was observed in 18-month-old mice [for 6-month-old, *t*_(46)_ = 2.64, *p* = 0.01; for 18-month-olds, *t*_(46)_ = 2.21, *p* = 0.03]. Although sex did not significantly influence the average amount of time required by DID and Water mice to complete the radial arm maze, we detected two significant 3-way interactions in which sex modified the time-course of learning over the first week of radial arm maze training. Deconstruction of the significant Sex × History × Session interaction [*F*_(5, 440)_ = 2.37, *p* = 0.04] along the sex factor did not detect any binge drinking effect in female subjects (Figure 12G, **left**) [Session effect: *F*_(5, 225)_ = 26.63, *p* < 0.0001; no drinking history effect or interaction, *p’s* > 30], while a significant interaction was detected for males (Figure 12G, **right**) [*F*_(5, 235)_ = 2.80, *p* = 0.02]. Male DID mice spent more and less time, respectively, than their Water controls to locate the platforms on sessions 3 and 5 of training [tests for simple effects, for session 3: *p* = 0.046; for session 5: *p* = 0.0006; for other sessions, *p’s* > 0.20]. Deconstruction of the significant Age × Sex × Session interaction [*F*_(5, 440)_ = 2.67, *p* = 0.02] along the Sex factor detected a main age effect for female subjects (Figure 12H, **left**) [*F*_(1, 45)_ = 14.13, *p* < 0.0001; interaction: *p* = 0.11], that reflected a longer latency to complete the maze in 18 vs. 6-month-old mice. For males, a significant Age × Session interaction was detected [*F*_(1, 48)_ = 22.23, *p* < 0.0001] that reflected a longer latency of 18-month-old males to complete the maze on sessions 2–6 (Figure 12H, **right**; tests for simple effects, *p’s* < 0.05; session 7, *p* = 0.21). These later findings reiterate the results presented in Figure 12F that both male and female 18-month-old mice require more time to navigate the radial arm maze than 6-month-old mice.

##### Chaining

An analysis of chaining behavior in Experiment 3 revealed a significant Age × History × Sex interaction [*F*_(1, 87)_ = 8.00, *p* = 0.006]. Deconstruction of the interaction along the Age factor indicated a History × Sex interaction for both 6 and 18-month-old mice [for 6-month-old, *F*_(1, 47)_ = 4.63, *p* = 0.04; for 18-month-old, *F*_(1, 47)_ = 4.00, *p* = 0.05]. For the 6-month-old mice (Figure 12I, **left**), the interaction reflected more chaining overall in DID females vs. their Water controls [for females, *t*_(22)_ = 2.89, *p* = 0.009; for males, *p* = 0.68]. In contrast, for the 18-month-old mice (Figure 12I, **right**), the interaction reflected non-significant trends for more chaining by females, but less chaining my males (*t*-tests, *p’s* = 0.21 and *p* = 0.12, respectively). We also detected a significant Age × Sex interaction for chaining [*F*_(1, 87)_ = 8.47, *p* = 0.005], that reflected more chaining by 18 vs. 6-month-old males (males in Figure 12I, **left vs. right**) [*t*_(47)_ = 2.34, *p* = 0.02], with a non-significant trend towards the opposite effect detected in females (*t*-test, *p* = 0.10), owing likely to the large binge drinking effect on chaining exhibited by 6-month-old females (females in Figure 12I, **left vs. right**) [*t*_(45)_ = 1.69, *p* = 0.10]. Although we detected a female- and age-selective effect of binge drinking on the average amount of chaining behavior exhibited by the mice in Experiment 3 (Figure 12I), neither sex nor age modified the effect of binge drinking on the time-course of chaining over the first week of radial arm maze training [History × Session: *F*_(5, 435)_ = 2.36, *p* = 0.04; other *p’s* > 0.12]. As illustrated in Figure 12J, this interaction reflected very distinct time-courses of chaining between DID and Water mice; while chaining behavior decreased linearly with training in Water mice [linear test for within-subjects contrast, *F*_(1, 47)_ = 8.86, *p* = 0.005], that of DID mice followed a quadratic function [linear test, *p* = 0.23; quadratic test: *F*_(1, 46)_ = 15.04, *p* < 0.0001], with DID mice exhibiting significantly more chaining behavior than Water mice on the 7th training session (tests for simple main effects, for session 7, *p* < 0.05; for other sessions, *p’s* > 0.05). These data provide additional evidence that mice with a history of binge drinking employ a non-memory navigational strategy to complete the radial arm maze.

## Discussion

AUDs are identified as the strongest modifiable risk factor for dementia onset (e.g., Schwarzinger et al., 2018). Based on concerns that: (1) the prevalence of AUD has increased more rapidly in women than in men over the past 10 years (Peltier et al., 2019), with mature adult females reporting a spike in heavy drinking during the first year of the COVID-19 pandemic (Pollard et al., 2020); and (2) women are nearly twice as likely to develop AD and related dementias than men (Hebert et al., 2013; Ferretti et al., 2018), we employed a mouse model of binge drinking to examine for sex differences in the cognitive effects of heavy drinking during mature adulthood and old age. In all, three experiments were conducted and a summary of their findings is compared in Table 2. Based on prior work indicating that the expression of alcohol withdrawal-induced negative affect in younger mice (<3 months of age) is age-dependent (e.g., Lee et al., 2016), we also examined for sex differences in the effects of binge drinking in later life on anxiety-like behavior during early withdrawal in Experiment 3 and a summary of those results are provided in Table 3.

**Table 2:** Comparison of the effects of a 1-month DID paradigm upon specific behavioral outcomes between Experiments 1 (heterogeneous cohort), 2 (6-month-old B6J mice), and 3 (6- and 18-month-old B6J mice).

**Dependent variable**	**Experiment 1**	**Experiment 2**	**Experiment 3**
**Binge drinking**
Total alcohol intake	F > M	F > M	F > M, 6 > 18
BECs	F = M	F > M	F < M, 6 < 18
**Morris Maze**
Latency to visible platform	DID > Water	DID > Water (females only)	DID > Water
Latency to hidden platform during training	DID = Water	DID = Water	DID > Water (females only; Session 1)
Latency to platform during probe test	DID = Water	DID > Water (females only)	DID > Water
Time spent in NE quadrant	DID = Water	DID = Water	DID = Water
Latency to new platform location	DID = Water	DID = Water	DID > Water (females only)
**Radial Arm Maze**
Days to acquisition criterion	DID = Water	DID > Water (females only)	DID > Water (6-month-old females only)
Reference Memory Errors	DID = Water	DID = Water	DID > Water (6 month-olds only)
Working Memory Correct Errors	DID = Water	DID = Water	DID > Water (6 month-olds only)
Working Memory Incorrect Errors	DID = Water	DID > Water (females only)	DID > Water (6 month-olds only)
Time to locate all platforms	DID = Water	DID = Water	DID > Water (6 month-olds) DID < Water (18 month-olds)
Chaining	Water = stable DID = U-shaped	DID > Water	DID > Water (6-month-old females for average)

*The sex- or age-specificity of DID effects is indicated in parentheses. F indicates female; M indicates Male. For Experiment 3, 6 indicates 6 month-old; 18 indicates 18 month-old*.

**Table 3:** Summary of the effects of a 1-month binge drinking (DID) paradigm upon specific behavioral outcomes during the behavioral test battery for negative affect conducted in the 6 and 18-month-old C57BL/6J mice of Experiment 3.

**Paradigm and Outcome**	**Drinking history**
**Acoustic Startle**	
Startle Amplitude	DID < Water (18 month-olds only)
PPI	DID = Water
**Light-Dark Box**	
Light side entries	DID < Water
Time in light side	DID < Water (18-month-old females only)
**Elevated Plus Maze**	
Time in open arm	DID < Water
Open arm entries	DID < Water
Closed arm entries	DID < Water (18-month-old males only)
**Novel Object Encounter**	
Latency to contact	DID > Water (females only)
Number of contacts	DID < Water
**Marble Burying**	DID > Water
**Forced Swim Test**	
Time immobile	DID < Water (6-month-old mice & 18-month-old males)
Number of immobile episodes	DID < Water

*The sex- and/or age-specificity of DID effects is indicated in parentheses. Graphical representations of these observations are presented in Figure 10*.

### Sex Differences in Binge Drinking Extend Into Old Age

To the best of our knowledge, no published study has reported on the biobehavioral consequences of voluntary binge drinking during the mature adult stage of development. That being said, one study by Grifasi et al. (2019) reported that old C57BL/6N mice (aged 18–20 months) will voluntarily binge drink alcohol to a similar extent as young adult (~2 months old) mice, with no evidence for sex differences in binge-intake. However, the Grifasi et al. (2019) study was under-powered to detect sex differences (*n* = 4 females). Acknowledging the obvious potential confounds associated with studying a heterogeneous group of mice in Experiment 1, we rationalized that if the sizes of any sex and/or alcohol effects were sufficiently large, they should be detectable, or at the very least, indicative of data trends worthy of follow-up investigation. Indeed, when adjusting for the co-variates of age and background, the results from Experiment 1 were largely negative (Table 2). However, they did demonstrate that mature adult mice engage in binge drinking, with females consuming more alcohol than males. Importantly, this sex difference in binge drinking was replicated in two independent studies controlling for the age of the subjects and their genetic/experiential background. Moreover, we also observed a sex difference in binge drinking by old mice (i.e., 18 months of age) in Experiment 3, which contrasts with the prior report by Grifasi et al. (2019). As female young adult (2-month-old) female C57BL/6NJ mice binge-drink more than males (Jimenez Chavez et al., 2021), this discrepancy in findings for older mice reflects the low female sample size employed in Grifasi et al. (2019), rather than the particular C57BL/6 substrain employed.

Estrogen is one important biomolecular driver of higher alcohol consumption by female mice (e.g., Becker and Koob, 2016; Guizzetti et al., 2016; Finn, 2020). However, the fact that a sex difference in binge drinking persists from early adolescence when mice are peri-pubertal (e.g., Finn et al., 2005; Strong et al., 2010; Jimenez Chavez et al., 2020) through to old age (present study) when female mice are in reproductive senescence (Flurkey and Harrison, 2007), argues strongly in favor of a more organizational, rather than activational, role for ovarian hormones in the stronger propensity of female subjects to binge-drink alcohol. Alternatively (or in concert with an organizational role for sex hormones), sex chromosomal factors may be also important contributors to sex differences in binge drinking. While this latter hypothesis has yet to be tested directly, sex chromosome complement influences sex differences in habitual responding to alcohol under operant-conditioning procedures (Barker et al., 2010), which has been posited to reflect sex chromosomal influences on synaptic plasticity-related genes within corticostriatal circuitry (Carruth et al., 2002; Chen et al., 2009; Jazin and Cahill, 2010). The fact that a sex difference in binge drinking persists across development has important implications for interpreting sex differences in the biobehavioral consequences of heavy alcohol-drinking—do females exhibit greater signs of AUD-related pathology because of their sex, or because they consume larger amounts of alcohol, or a combination of both factors? Such a question highlights the need for studies that disentangle biological sex from sex-related differences in alcohol intake to better understand how these subject factors impact the short- and longer-term consequences of heavy drinking.

### Alcohol Withdrawal Elicits a Negative Affective State in Older Mice

Another consistent finding across all three experiments in this report is the observation that mature adult DID mice exhibit impaired visually-cued navigation upon first exposure to the Morris water maze. While this effect was apparent only in the female subjects of Experiment 2, no sex difference was detected for this variable in the other two studies of 6-month-old mice. Although 18-month-old females were slower overall to locate the flagged platform than males, the alcohol-induced impairment in platform location was also not sex-selective. This argues that the sex-selectivity of the Experiment 2 result may be spurious, particularly considering that Experiment 3 was sufficiently powered to detect sex differences in other cognitive outcomes (see Table 2). The deficit in visually-cued navigation during the Flag Test might reflect poorer attention or visual acuity in DID mice; however, the DID mice in all three experiments successfully employed intra-maze visual cues to learn the locations of the hidden platforms within both the Morris water and radial arm mazes, arguing against a problem with visual attention or acuity. The alcohol-induced deficit in Flag Test performance also did not likely reflect an incapacity to swim or excessive floating behavior, as DID mice in all three studies swam a longer distance during the Flag Test than the Water controls.

In younger adult B6J mice (i.e., 2 months of age), binge drinking induces a robust negative affective state that is apparent at 24 h into alcohol withdrawal (e.g., Lee et al., 2015, 2016; Jimenez Chavez et al., 2020). The persistence of this withdrawal-induced negative affective state increases as a function of the duration of the drinking period, with heightened indices of photophobia, neophobia, and agoraphobia, as well as increased “panic-like” swimming in the forced swim test detected for up to 30 days in mice with month-long period of binge drinking (Lee et al., 2015), such as that employed in the present study. To the best of our knowledge, there is very limited study of how the severity of the psychophysiological state during early alcohol withdrawal (a.k.a. “hang-over”) changes with older age. Thus, we rationalized that the alcohol-related performance deficit on the Flag Test might reflect alcohol withdrawal-induced hyper-reactivity to swim stress. This possibility was supported by the results of the behavioral screen in Experiment 3, in which both 6 and 18-month-old DID mice exhibited clear signs of withdrawal-induced negative affect, with increased swimming in the forced swim test observed in concert with more traditional behavioral indices of anxiety-like behavior (Table 3). As reported for young adults (Jimenez Chavez et al., 2020), the magnitude of this negative affective state was largely sex-independent, with the majority of anxiety-related dependent variables similarly impacted by binge drinking in mature adult and old mice (see Table 3). Such findings support the notion that the performance deficit during the Flag Test exhibited by all of the DID mice in this study might simply reflect behavioral hyper-reactivity to being placed in a relatively large tub of water, which interfered with or overrode their ability to focus on searching for a means of escape.

This being said, 18-month-old females did not exhibit any alcohol-induced change in swimming behavior in the forced swim test (Table 3). However, these mice exhibited robust signs of withdrawal-induced anxiety in every other assay in the behavioral screen, clearly indicating that they experience a negative affective state in alcohol withdrawal that could very well have manifested as hyper-reactive swimming during the Flag Test. These findings are consistent with a prior study by Novier et al. (2016), indicating that chronic, intermittent, alcohol consumption by young adult and aged (18+ month-old) rats induces comparable signs of withdrawal-induced negative affect in the elevated plus maze. However, the present data diverge from another study in which adolescent, adult, and aged rats failed to exhibit any signs of negative effects following a history of repeated alcohol injections (Grifasi et al., 2019). While we have yet to directly compare the effects of repeated alcohol injections to voluntary binge drinking on the severity of withdrawal-induced negative effect, repeated injections of alcohol (15 injections of 4 g/kg, every other day) to young adult B6J mice induce a relatively weak negative affective state (Fultz et al., 2021), compared to that observed following 14 consecutive days of binge drinking (Lee et al., 2016; Jimenez Chavez et al., 2020), as indexed by the total number of specific variables impacted by alcohol history. Thus, the discrepancies in findings between studies examining high-dose alcohol drinking in older rodents (Novier et al., 2016; present study) and those employing alcohol injection regimens (Grifasi et al., 2019) likely reflect differences in alcohol pharmacokinetics related to the dose administered, route of administration or intermittency of administration rather than an age-related decline in sensitivity to withdrawal-induced negative effect.

### Anxiety-Like Behavior Does Not Vary Systematically With Age

An age-related change in anxiety-like behaviors is reported to occur in B6 mice, notably in paradigms similar to those employed in the present study (Shoji et al., 2016; but see Malatynska et al., 2012). However, akin to a prior report (Malatynska et al., 2012) and as highlighted above, we detected no age-related difference in baseline anxiety-like behavior in Water controls or in the magnitude of alcohol’s effect on anxiety-like behavior in DID mice for the vast majority of our anxiety-related dependent variables (Table 3). One notable exception was the relatively long time spent in the light-side of the light-dark shuttle-box by female 18-month-old Water controls. More time spent in the light-side is typically interpreted as reflecting hypo-anxiety (Karl et al., 2003; Walf and Frye, 2007) and consistent with the possibility that old female mice may be relatively hypo-anxious, the 18 month-old Water females in the present study exhibited the least amount of marble-burying of all of the groups tested. Although 18 + month-old mice were not included in their study, Shoji et al. (2016) reported more open arm exploration by 7–9 month-old B6 mice, relative to younger adult mice (≤5 months old) in an elevated plus-maze. In contrast, we did not detect any age-related difference in open arm time or entries in our study of 6 vs. 18-month-old animals, which might reflect the fact that we did not include younger mice or that these different behavioral indices reflect different aspects of anxiety-like behavior, as demonstrated by others (Belzung and Le Pape, 1994; Milner and Crabbe, 2008).

In addition to some signs of hypo-anxiety, we also noted that 18-month-old female Water controls exhibited significantly fewer closed arm entries in the elevated plus maze, relative to their younger counterparts. Although not as precise a measure of locomotor activity as the distance traveled in an open field, this observation raises the alternate possibility that their lower marble-burying behavior and/or longer time spent in the light-side of the light-dark box might simply reflect generalized hypoactivity. Indeed, a number of studies have demonstrated an age-dependent reduction in general locomotor activity expressed by B6 mice when placed in novel environments (Francia et al., 2006; Benice et al., 2006; Lalonde and Strazielle, 2009; Shoji et al., 2016), although locomotor hyperactivity is reported in 18–20 month-old B6 mice when their behavior is assayed near the end of the dark phase of their circadian cycle—phenomenon consistent with “sundowning syndrome” (Bedrosian et al., 2011). When examined, the age-related changes in general locomotor activity are sex-independent (Benice et al., 2006). In stark contrast to their female counterparts, the 18-month-old male Water controls in our study were significantly *more* active than 6-month-old males in the elevated plus maze (indexed by closed arm entries). As all of the mice in the present study were tested over the first 5–6 h of the 12-h dark phase, it is not likely that the hyperactivity of the 18-month-old males expressed in the elevated plus maze reflects “sundowning syndrome”. Taken all together, the results of the present study and those of prior behavioral screens of older mice (David et al., 2001; Kasckow et al., 2005; Godbout et al., 2008; Moretti et al., 2011; Malatynska et al., 2012; Shoji et al., 2016), do not indicate a consistent effect of aging on behavioral measures of anxiety-like behavior.

### Binge Drinking During Mature Adulthood Induces Many Female-Selective Signs of Cognitive Impairment

Controlling for age at drinking and genetic background, we found that a month-long history of binge drinking negatively impacted several cognitive measures in mature adult mice but with different variables affected across Experiments 2 and 3 (Table 2). While the incongruence in findings may be considered empirically problematic, behavior is inherently variable, even when studying isogenic mice of the same age (e.g., Wahlsten et al., 2003; Bohlen et al., 2014). Thus, it should not be expected that every single finding replicates perfectly across studies, particularly when the studies are conducted nearly a year apart in time and/or by different groups of investigators (see Bohlen et al., 2014), as was the case with the present work. In our opinion, more noteworthy is the fact that binge drinking impacted half of our cognition-related dependent variables in Experiment 2 and impairments were detected under both Morris water and radial arm maze procedures. Thus, impairments were apparent across two paradigms that tap into overlapping, but distinct, cognitive and neural processes (e.g., Rossi-Arnaud and Ammassari-Teule, 1994; Floresco et al., 1997, 1999; D’Hooge and De Deyn, 2001; Vorhees and Williams, 2014).

Further, when the statistical power of the study was increased by the inclusion of the older mice, all but one of our dependent variables in Experiment 3 demonstrated a binge drinking effect in mature adult mice and the direction of *all* alcohol effects in the mature adult DID mice were indicative of poorer cognition. Further, in all three experiments, DID mice exhibited a high level of “chaining” behavior in the radial arm maze, indicating that these mice employed a non-cognitive strategy to successfully navigate the maze. Adding to this, a direct comparison of the behavior exhibited by the 6-month-old DID mice and that of 18-month-old Water controls confirmed that the magnitude or “severity” of the drinking-induced cognitive impairments is comparable to that exhibited by an alcohol-naïve more senior animal. By definition, such findings indicate that a history of binge drinking during mature adulthood is sufficient to accelerate cognitive decline and future work seeks to determine whether this acceleration reflects an alcohol-induced up-regulation of protein changes and neuropathologies associated with AD and related dementias, as has been reported to occur in both transgenic models of AD vulnerability (Hoffman et al., 2019; Ledesma et al., 2020) and wild-type rodents (Salling et al., 2016; Agoglia et al., 2017; Grifasi et al., 2019; Hoffman et al., 2019; Van Hees et al., 2022).

Another cognition-related finding that replicated across Experiments 2 and 3 was the female-selective increase in the number of training days required by mature adult mice to reach the acquisition criterion in the radial arm maze (Table 2). Not shown herein, a cursory analysis of the unadjusted means from Experiment 1 also indicated a significantly longer latency for female mice to acquire the radial arm maze than males, but this sex difference was lost when the data were adjusted for age and background. Indeed, when background and age were controlled and age factored into the analysis of the radial arm maze data in Experiment 3, no sex differences were detected in 18-month-old mice, suggesting that age of drinking-onset is an important variable affecting the ability to detect sex differences in alcohol’s effects on this particular variable. Although the specific variable affected by alcohol varied across Experiments 2 and 3, it is noteworthy that nearly half of the variables exhibited a female-selective effect, with the level of impairment comparable to more aged female controls. Arguably more noteworthy, no male-selective effects of alcohol were detected in any study (Table 2). Recognizing that Experiments 2 and 3 were conducted nearly a year apart in time, the sum of the results from these two studies argues that female mature adult mice are more sensitive to alcohol-induced cognitive impairments and these cognitive impairments manifest under both Morris water maze and radial arm maze procedures. As no sex difference is observed for swimming behavior by mature adult DID mice in the forced swim test (Table 3), this sex difference in alcohol-induced cognitive impairment in these water-based assays does not likely reflect differential sensitivity to swim stress. Female humans and laboratory rodents are reported to exhibit poor spatial navigation than males (e.g., Brandeis et al., 1989; Williams and Meck, 1991; Beatty, 1992; Bimonte et al., 2000; Grootendorst et al., 2004) and to exhibit a faster rate of cognitive decline when compared on visuo-spatial tasks than males (Small et al., 1995; King et al., 1999; Markowska, 1999; Frick et al., 2000; Benice et al., 2006). In the present study, 6-month-old females required more time than males to locate the hidden platform on the first Morris water maze training session in both Experiments 2 and 3. Such sex differences in visuo-spatial learning have been argued to limit the ability to detect sex differences in the effects of experimental manipulations (transgenic or pharmacological) on spatial navigation measures (see Benice et al., 2006) and may very well have masked our ability to detect more female-selective results herein. As the majority of studies focused on the cognitive-impairing effects of alcohol have excluded female subjects or were underpowered to detect sex differences (e.g., Slawecki, 2006; Kuzmin et al., 2012; Broadwater and Spear, 2014; Badanich et al., 2016; Novier et al., 2016; Fernandez et al., 2016; Hoffman et al., 2019; Ledesma et al., 2020), it will be important to extend our results for mature adult mice to land-based paradigms that do not involve spatial navigation and determine whether the sex selectivity of any alcohol-related cognitive decline is reflected by specific neuropathology.

### The Effects of Binge Drinking During Old Age Are Paradigm-Dependent

The statistical results of Experiment 1 indicated that age significantly impacted our ability to detect the effect of binge drinking on cognitive performance in both the Morris water and radial arm mazes. Based on these results and prior work indicating that a history of binge drinking during old age (18+ months) induces anomalies in hippocampal microglia that are not observed in adolescent binge drinking mice (Grifasi et al., 2019), we included 18-month-old mice in our study of alcohol-induced cognitive decline. As expected, based on the mouse aging literature (e.g., Benice et al., 2006; Zhao et al., 2011; Shoji et al., 2016), we detected several signs of age-related cognitive impairment in Experiment 3, irrespective of alcohol history. In the Morris water maze, 18-month-old mice took longer to locate the flagged platform and to reach the former platform location on the probe test. In the radial arm maze, 18-month-old males took longer than their 6 month-counterparts to acquire the maze and 18-month-old mice of both sexes made more reference memory errors, working memory correct and incorrect errors, and required more time to complete the radial arm maze during the first week of training. Thus, a number of our dependent variables were sensitive not only to sex and alcohol but also to age.

The 18-month-old mice in the present study consumed less alcohol than their younger counterparts but achieved a BEC within error of the 80 mg/dl criterion for binge drinking (National Institute on Alcohol Abuse and Alcoholism, 2007). The age difference in alcohol consumption observed herein aligns with that reported in a prior study of male 2.5 and ~18-month-old rats fed a liquid alcohol diet (Novier et al., 2016) and is consistent with an age-related slowing of alcohol metabolism (e.g., Collins et al., 1975; Oneta et al., 2001; Kim et al., 2003). Also consistent with Novier et al. (2016), we detected no age-related differences in the negative impact of heavy drinking on Morris water maze performance (Table 2). Together, these data indicate that heaving drinking during old age is sufficient to exacerbate impairments in spatial learning and memory in both rat and mouse models of voluntary excessive drinking that are consistent with the clinical literature (Thomas and Rockwood, 2001; McMurtray et al., 2006; Weyerer et al., 2011; Grant et al., 2017; Xu et al., 2017; Huang et al., 2018; Sabia et al., 2018; Schwarzinger et al., 2018).

To our surprise, we failed to detect any alcohol-induced impairment in the radial arm maze performance of 18-month-old mice. In fact, the only alcohol effect detected for 18-month-old mice was a quickening of the time taken to locate all 4 hidden platforms during maze training (Table 2)—an effect opposite that predicted from their higher number of reference and working memory correct errors. Although a ceiling effect might exist for reference memory errors as animals can only commit four such errors per session, we cannot discern from the present experimental design whether or not a ceiling effect might exist also for working memory-related errors, given the 2-min duration of each training session. Perhaps a longer training session may afford more opportunity for older DID mice to commit more working memory errors? However, as the number of errors committed by the 18-month mice declined over the week of training, a ceiling effect cannot account for negative results during later training sessions when the number of errors was relatively low. As mentioned above, Morris water maze and radial arm maze recruit distinct neural networks. Although the intact hippocampal function is required for both tasks (e.g., Floresco et al., 1997; Vorhees and Williams, 2014), the Morris maze requires the more medial aspect of the dorsal striatum, basal forebrain, cerebellum, and insula (D’Hooge and De Deyn, 2001), while the radial arm maze also requires the medial prefrontal cortex, ventral subiculum, mediodorsal thalamus, and amygdala (Rossi-Arnaud and Ammassari-Teule, 1994; Floresco et al., 1997, 1999). Thus, the distinct possibility exists that, despite binge drinking alcohol for a month, this alcohol history was insufficient to exacerbate extant age-related perturbations in the function of these latter brain regions.

Indeed, it is worth noting that in none of our three experiments did we detect any effect of binge drinking or age on reversal learning in the Morris water maze (Table 2). Reversal learning requires the ventrolateral prefrontal cortex and lateral orbitofrontal cortex (Iversen and Mishkin, 1970; Schoenbaum et al., 2002; Chudasama and Robbins, 2003) and activates a number of other cortical areas including the inferior frontal gyrus (Nagahama et al., 2001; Cools et al., 2002), the dorsomedial and dorsolateral prefrontal cortex (Remijnse et al., 2005; Budhani et al., 2007; Mitchell et al., 2009), the posterior parietal cortex (Hampshire and Owen, 2006; Gläscher et al., 2009), in addition to the striatum (Rogers et al., 2000; Cools et al., 2002; Hampton and O’Doherty, 2007; Clarke et al., 2008; Mitchell et al., 2008; Tanaka et al., 2008). Binge drinking is reported to impair the function of the limbic system, diencephalon, frontal, and middle and inferior temporal lobes and hyper-actives the right superior frontal and parietal lobes (c.f., Waszkiewicz et al., 2018), with even a single binge drinking session sufficient to cause gliosis in the piriform, entorhinal, and perirhinal cortices, as well as the dentate gyrus (Hayes et al., 2013). Whether or not this specific binge-induced neuropathology or other neuropathology associated with normal aging or neurodegeneration map to impairments in specific cognitive domains is a major research question that cannot be addressed in a single study. Our study indicates that sex and the age at binge drinking onset are two factors that can modify the impact of alcohol on visuo-spatial navigation, reference, and working memory, thereby providing a solid foundation for future work aimed at understanding how biological sex interacts with alcohol history to alter the trajectory of age-related cognitive decline and associated neuropathology.

## Data Availability Statement

The raw data supporting the conclusions of this article will be made available by the authors, without undue reservation.

## Ethics Statement

The animal study was reviewed and approved by Institutional Animal Care and Use Committee of the University of California Santa Barbara.

## Author Contributions

KS and CJ: conceptualization and supervision. KS: formal analysis, writing—original draft preparation, visualization, and project administration. KS, EVD, JM, NO, AK, LM, IK, JH, JT-G, ER, and CJ: investigation, writing—review and editing. KS, CJ, IK, LM, and NO: funding acquisition. All authors contributed to the article and approved the submitted version.

## Conflict of Interest

The authors declare that the research was conducted in the absence of any commercial or financial relationships that could be construed as a potential conflict of interest.

## Publisher’s Note

All claims expressed in this article are solely those of the authors and do not necessarily represent those of their affiliated organizations, or those of the publisher, the editors and the reviewers. Any product that may be evaluated in this article, or claim that may be made by its manufacturer, is not guaranteed or endorsed by the publisher.
